# Functional photosystem I maintains proper energy balance during nitrogen depletion in *Chlamydomonas reinhardtii*, promoting triacylglycerol accumulation

**DOI:** 10.1186/s13068-017-0774-4

**Published:** 2017-04-13

**Authors:** Mahmoud Gargouri, Philip D. Bates, Jeong-Jin Park, Helmut Kirchhoff, David R. Gang

**Affiliations:** 1 Institute of Biological Chemistry, Washington State University, Pullman, WA 99164 USA; 2 Laboratory of Plant Molecular Physiology, Center of Biotechnology of Borj Cedria, P.O. Box 901, 2050 Hammam-Lif, Tunisia; 3 Department of Chemistry and Biochemistry, The University of Southern Mississippi, Hattiesburg, MS 39406 USA

**Keywords:** Photosystem I, TAB2, *Chlamydomonas reinhardtii*, Nitrogen depletion, Triacylglycerol, Omics analysis

## Abstract

**Background:**

Nutrient deprivation causes significant stress to the unicellular microalga, *Chlamydomonas reinhardtii*, which responds by significantly altering its metabolic program. Following N deprivation, the accumulation of starch and triacylglycerols (TAGs) is significantly altered following massive reprogramming of cellular metabolism. One protein that was found to change dramatically and early to this stress was TAB2, a photosystem I (PSI) translation initiation factor, whose transcript and protein levels increased significantly after only 30 min of N deprivation. A detailed physiological and omics-based analysis of an insertional mutant of Chlamydomonas with reduced TAB2 function was conducted to determine what role the functional PSI plays in regulating the cellular response to N deprivation.

**Results:**

The *tab2* mutant displayed increased acetate assimilation and elevated starch levels during the first 6 h of N deprivation, followed by a shift toward altered amino acid synthesis, reduced TAG content and altered fatty acid profiles. These results suggested a central role for PSI in controlling cellular metabolism and its implication in regulation of lipid/starch partitioning. Time course analyses of the *tab2* mutant versus wild type under N-deprived versus N replete conditions revealed changes in the ATP/NADPH ratio and suggested that TAG biosynthesis may be associated with maintaining the redox state of the cell during N deprivation. The loss of ability to accumulate TAG in the *tab2* mutant co-occurred with an up-regulation of photo-protective mechanisms, suggesting that the synthesis of TAG in the wild type occurs not only as a temporal energy sink, but also as a protective electron sink.

**Conclusions:**

By exploiting the *tab2* mutation in the cells of *C. reinhardtii* cultured under autotrophic, mixotrophic, and heterotrophic conditions during nitrogen replete growth and for the first 8 days of nitrogen deprivation, we showed that TAG accumulation and lipid/starch partitioning are dynamically regulated by alterations in PSI function, which concomitantly alters the immediate ATP/NADPH demand. This occurs even without removal of nitrogen from the medium, but sufficient external carbon must nevertheless be available. Efforts to increase lipid accumulation in algae such as Chlamydomonas need to consider carefully how the energy balance of the cell is involved in or affected by such efforts and that numerous layers of metabolic and genetic regulatory control are likely to interfere with such efforts to control oil biosynthesis. Such knowledge will enable synthetic biology approaches to alter the response to the N depletion stress, leading to rewiring of the regulatory networks so that lipid accumulation could be turned on in the absence of N deprivation, allowing for the development of algal production strains with highly enhanced lipid accumulation profiles.

**Electronic supplementary material:**

The online version of this article (doi:10.1186/s13068-017-0774-4) contains supplementary material, which is available to authorized users.

## Background

Microalgae-derived biodiesel is a promising next generation biofuel due to its sustainability and biodegradability. Under certain conditions, such as nitrogen deprivation, strains of microalga such as *Chlamydomonas reinhardtii* are capable of producing significantly elevated levels of high-value compounds, such as triacylglycerols (TAGs) [1], which can be converted into multiple valuable fuels such as biodiesel, gasoline, and jet fuel [2]. Plant oils are the most energy dense form of biological energy storage [3]. Increasing the conversion of photosynthates into oils presents a potentially cost-efficient way to capture and store solar energy [4]. However, increasing oil production in microalgae and plants involves overcoming significant challenges [5]. The massive TAG accumulation that can occur in microalgae during nitrogen deprivation is correlated with a reduced abundance of many transcripts and proteins involved in photosynthesis, carbon fixation, and chlorophyll synthesis [6–8]. This leads ultimately to cessation of cell division and stalled growth. Therefore, an enhanced understanding of the relationship between maintaining active photosynthesis and high lipid accumulation will be required in order to rationally engineer the regulatory networks that control photosynthetic output in microalgae.

A number of other studies have focused on understanding the relationship between starch and lipid metabolism through examination of various Chlamydomonas wild-type strains and starchless mutants [9–11]. While it is clear that carbon accumulation switches from starch to lipid during N depletion, the role of energy production by PSI and PSII during the sensation of growth and TAG accumulation is still unclear. The light reactions of photosynthesis that produce energy and reducing equivalents (ATP and NADPH) for fatty acid synthesis may be a limiting factor with regards to TAG synthesis capability [12, 13]. Therefore, a better understanding of the mechanisms that regulate photosynthesis under nutrient starved (e.g., N deprivation) conditions is urgently needed.

Accumulation of carbon as TAG during N depletion demands much more reductant than accumulation of carbon as starch during N replete conditions. However, many previous studies suggest that changes in electron flow through PSI and PSII during N depletion do not favor production of NADPH for fatty acid synthesis. Several studies have used fluorescence measurements to begin unraveling the molecular mechanisms of photosynthesis inhibition in microalgae and diatoms, leading to the conclusion that photosystem II (PSII) is more highly affected than photosystem I (PSI) in response to nitrogen limitation [14, 15]. Systems biology approaches have been employed to understand the changes within the photosynthetic apparatus during N deprivation and have revealed an overall down-regulation of the majority of transcripts related to PSII and PSI after 2 days of N depletion [7, 16]. A quantitative proteomic study of starved-wild-type cells [17] demonstrated that down-regulation of PSII was coupled to up-regulation of PSI, suggesting that there is a relatively large capacity for cyclic electron (CEF) flow under N deprivation. Recently [8] demonstrated that linear electron flow (LEF) fell ~15% more than CEF over the first 24 h of N deprivation. The increase of CEF capacity implies that there is an increase in demand for photosynthetically generated ATP relative to NADPH. However, lipid biosynthesis has a high NADPH demand [18]. The decreased PSII content and the linear electron transport under nitrogen deprivation raise the question of how lipid accumulation is initiated and maintained although photosynthetic NADPH production is reduced. It has been suggested that several mechanisms of alternative electron acceptor pathways, such as chlororespiration, are involved in regulating the ATP/NADPH ratio and photosynthetic output during the first 24 h of N deprivation [8]. Thus, a better understanding of ATP/NADPH production during N deprivation is needed. Clearly, specific regulatory proteins and perhaps signaling pathways are involved to maintain proper ATP and NADPH production for fatty acid synthesis under these conditions.

To identify these specific regulators, we previously analyzed the expression profiles of 417 putative regulatory genes in Chlamydomonas, including those involved in the regulation of transcription and translation, beginning from the onset of nutrient removal from the media through initiation of lipid accumulation. Several such regulatory proteins were found to respond to the nutrient stress. Among these, one stood out as an early and strong responder, the *TAB2* gene [19]. TAB2 has been reported to be an RNA-binding protein that plays a key role in the initial steps of psaB translation and PSI assembly under normal nutrient conditions [20]. The F14 mutant strain of *C. reinhardtii* CC-1044 harbors a 14 nucleotide insertion near the 3′ end of the *TAB2* gene, eliminating a highly conserved tryptophan at position 348, which is required for the TAB2 protein to function properly in initiating psaB translation [20]. The *tab2* mutant displays reduced psaB translation and a concomitant reduction in PSI assembly.

Therefore, to unveil the relationship between photosynthesis and TAG accumulation in algae, we carried out a detailed characterization of the *tab2* mutant and evaluated changes in the metabolome and proteome, as well as in photosynthetic parameters, relative to the CC-1044 wild type within growth time courses in N replete versus N-depleted growth conditions.

## Methods

### Strain, culture conditions, and sampling

Two Chlamydomonas strains were used in this investigation. The wild-type strain, CC-125 wild-type mt+ [137c], was obtained from the Chlamydomonas Genetics Center at Duke University (http://chlamycollection.org/) and the *tab2* mutant strain (F14 mt+) was kindly provided by the Institut de Biologie Physico-Chimique (IBPC), France. Both strains were grown at 25 °C in continuous light (70 μmol photons m^−2^ s^−1^) in the presence of acetate in liquid cultures under shaking (150 rpm). Standard tris–acetate-phosphate (TAP) medium, which included 7.5 mM NH_4_Cl, and high salt (HS) medium were previously described [21, 22]. For photoautotrophic growth, mutant cells were able to grow only on liquid culture when humidified ambient air was circulated through the culture flasks to increase CO2 availability. For nitrogen starvation studies, exponential phase (4 × 10^6^ cells ml^−1^) cultures were centrifuged at 1000×*g* for 5 min at room temperature, cell pellets kept and washed twice in TAP or HS medium either with or without nitrogen (TAP-N) and (HS-N). Pellets were then resuspended in medium without nitrogen and cells were grown under constant light with shaking. Samples for analysis were taken immediately after resuspension (time 0) or periodically during the growth time courses, and were pelleted as outlined above. Culture growth was monitored by counting cells with a hemocytometer [21]. Cell size was assessed using a Leica TCS SP5 laser-scanning confocal microscope with the assumption that cells are spherical for diameter calculations. Cells were immobilized with the addition of 10 µl of 3 M potassium iodide in 1 ml algal culture medium and kept for 10 min before microscopy analysis.

### Quantitative RT-PCR conditions and analysis

Total RNA was isolated from the cell pellet using the Trizol reagent (Ambion). One microgram of total RNA was used as a template for each RT-reaction following the manufacturer’s instructions (qScript cDNA SuperMix, Quanta Bioscinces). Gene-specific primers were designed to amplify fragments of approximately 100–150 bp in length. For the quantification of gene expression, qPCR was carried out on a Mastercycle Realplex 2 (Eppendorf) using the Perfecta Syber Green Fast Mix (Quanta Bioscinces, Gaithersburg, MD, USA). The actin gene served as internal control for the quantification assays in *C. reinhardtii*. For gene expression analysis by qPCR, the expression values were calculated according to the 2−∆∆CT method (for each gene, ∆CT = CT, Gene − CT, housekeeping specific). The ∆∆Ct calculation was validated using the plot of the log cDNA dilution versus ∆Ct. See supplemental material (Additional file 1: Table S1) for all primer sequences used in this work.

### Proteomic analysis

*Chlamydomonas* strains were harvested by centrifugation at 3000×*g* for 5 min at 4 °C. Proteins were extracted from 50–100 mg of cells as described previously [23] and were quantified using the Qubit Protein Assay Kit (Invitrogen, Carlsbad, USA) in accordance with the supplier’s protocol for the Qubit 2.0 fluorometer (Invitrogen). 100 µg of each sample was digested with trypsin and analyzed on an Orbitrap Fusion Tribrid mass spectrometer (Thermo Scientific, Rockford, USA) coupled with an EASY-nLC (Thermo Scientific). Data were processed and searched using SIEVE 2.1 (Thermo Scientific), and all searches were performed against the Chlamydomonas protein database from Phytozome v. 9.0 (http://www.phytozome.net) and NCBI chloroplast (http://www.ncbi.nlm.nih.gov/nuccore/BK000554) and mitochondrion (http://www.ncbi.nlm.nih.gov/nuccore/NC_001638.1) databases.

### Metabolomic analysis

The relative levels of amino and organic acids and sugars were determined by gas chromatography-mass spectrometry (GC–MS). The lyophilized cells (5 ml of culture pelleted as above) were transferred to a 1.5-ml eppendorf tube and disrupted using a steel ball (5 mm). To each sample, 0.75 ml of extraction buffer (methanol:chloroform:water, 5:2:2 v/v) was added. Samples were then vortexed for 10 min at room temperature, followed by centrifugation at 16,000×*g* for 2 min. The supernatant was collected, evaporated to dryness and spiked with 2 µl of internal standard mixture solution of methyl esters and C30 linear chain length fatty acids. After 5 µl pyridine methoxyamine hydrochloride was added, each sample was incubated for 90 min in a thermostatic bath kept at 30 °C. Samples were then derivatized by the addition of 45 µl *N*-methyl-*N*-trimethylsilyltrifluoroacetamide (MSTFA + 1%TMCS, Pierce, Rockford, IL, USA) for 30 min at 37 °C. Derivatized primary metabolites were analyzed by gas chromatography-time of flight mass spectrometry (GC-TOFMS) on a Pegasus 4D GC–MS system (LECO, St. Joseph, MI, USA) equipped with an RTX^®^-5Sil MS with Integra-Guard^®^ column (30 m × 0.25 mm ID × 0.25 μm film thickness) from Restek (GmbH, Bad Homburg, Germany) and an MPS-2 autosampler (Gerstel, Muehlheim, Germany). All injections were performed in splitless mode (1 µl) with helium as carrier gas at a constant flow of 1 ml min^−1^. The column was held isothermally at 50 °C for 1 min and ramped at 20 °C min^−1^ to 330 °C. ChromaTOF software version 4.41 equipped with the LECO/Fiehn Metabolomics database was used for primary metabolite data processing.

### Lipid analysis

#### Total lipid analysis

A simplified and modified protocol using direct transmethylation [24] was applied to microalgal cultures for high-speed screening purposes. Briefly, 5 million cells were harvested by centrifugation at 10,000×*g* for 2 min. The cells were freeze-dried and extracted with 1 ml of 2.5% (v/v) conc. sulfuric acid in methanol, with 10 μg of C15:0 TAG used as a triacylglycerol internal standard. The transesterification reaction was allowed to occur at 80 °C for 60 min. After cooling to room temperature, 0.2 ml of hexane and 1.5 ml of water were added and vortexed. The organic phase was separated from the water phase by centrifugation at 4000×*g* for 2 min. The resulting fatty acid methyl esters (FAMEs), present in the organic phase, were analyzed by gas chromatography coupled to a flame ionization detector (FID) using an EC Wax column (30 m, 0.53 mm i.d., 1.20 µm film thickness; Alltech). The GC conditions were as follows: split mode injection (1:40), injector and flame ionization detector temperature: 260 °C; oven temperature program: 190 °C for 2 min, then increasing at 10 °C min^−1^ to 250 °C and holding this temperature for 5 min.

#### Neutral lipid analysis

Neutral lipids were extracted using a procedure modified from [25]. Cells were collected by centrifugation at 10,000×*g* for 10 min and were resuspended in 0.7 ml of 1 mM EDTA in 0.15 M acetic acid. This solution was vortexed for 10 min after the addition of 3 ml methanol:chloroform (2:1, v/v). Then, 1.5 ml of chloroform and 1.2 ml of KCl 0.88% (w/v) were added. After vortexing, the phases were separated by centrifugation at 2000×*g* for 2 min. The lower, chloroform phase was removed to a clean tube using a glass transfer pipette. The upper, aqueous phase was re-extracted with an additional 2 ml of chloroform. This solution was vortexed and centrifuged as before. The lower organic layer was then combined with that obtained in the first extraction and the total lipid extract was taken to dryness under a gentle stream of nitrogen. Lipids were redissolved in 200 µl of chloroform for further analysis. Triacylglycerols (TAGs) were separated from other lipids using thin layer chromatography (TLC). Typically, around 0.5 mg of lipid extract was loaded as a spot onto a 20 × 20 cm Partisil K6 silica gel 60 Å TLC plate (Whatman, Maidstone, UK). Plates were then developed using hexane/diethyether/acetic acid (80/20/2, v/v/v) for 30 min. The TAG fraction was determined based on migration of standards, and was visualized under UV light after the plate was sprayed with a fine mist of 0.01% primuline in acetone:H_2_O (4:1). The silica corresponding to the TAG fraction was carefully scrapped off of the TLC plate and was transmethylated for GC-FID analysis as described above. However, the recovery of TAG from the silica, transmethylation, and quantification of the FAMEs by GC-FID is a time consuming method. A solid-phase extraction method using Discovery DSC-Si SPE cartridges (Supelco, Bellefonte, PA, USA) was developed for high throughput analysis of the neutral lipid fraction. Cartridges containing 100 mg of solid phase were conditioned with 6 ml of methanol and then 6 ml of chloroform. The samples (200 µl in chloroform) were loaded onto the cartridges using a syringe. The neutral lipid fraction was eluted with 1 ml chloroform:methanol (99:1). This fraction contained the TAGs and DAGs while the polar lipid fraction was retained on the cartridge. The latter was eluted with 1 ml chloroform:methanol (2:1). The neutral fraction was taken to dryness under a gentle stream of nitrogen and the FAMEs were generated and quantified by GC-FID as described above. Results obtained by the solid-phase extraction method were not significantly different from those obtained by recovery of the TAG fraction from the TLC plate.

### Starch, chlorophyll quantification, and cell viability

Total starch was quantified using an enzymatic starch assay kit including a commercial amyloglucosidase solution (Sigma SA-20) to convert starch to glucose (Sigma-Aldrich, St. Louis, MO, USA). A total of 4 million cells were harvested by centrifugation for 2 min at room temp. The pellet was resuspended in 1 ml 80% acetone and mixed vigorously to extract chlorophyll. After centrifugation at high speed for 1 min, the supernatant was used to determine the total chlorophyll content based on the absorbance values at 663 and 645 nm following Arnon’s method [26]. The resulting pellet was taken to dryness in a fume hood and then resuspended in 400 µl of water. Total starch content was measured using a standard method [27] after the starch had been solubilized by autoclaving the resuspended pellet for 15 min at 120 °C. To assess cell viability, cells grown in TAP-N medium were harvested at different time points in the growth time courses (0, 12, 24, 48, 96, and 144 h). For each time point, 0.1 ml of the culture was diluted 1:100 and then 0.1 ml of the diluted culture was spread on a TAP agar plate and allowed to grow for 1 week. Viability percentages corresponded to the total number of colonies counted for each time point relative to the 0-h time point as described by [28].

### Oxygen evolution and photosynthesis parameters

Steady-state rates of oxygen evolution in nitrogen-deprived cells were determined using a water-jacketed (20 °C) Clark-type oxygen electrode (Oxylab, Hansatech Instruments, King’s Lynn, UK). Aliquots of cells (1 ml) were withdrawn from the flasks and dark adapted for 20 min before being transferred to the measuring chamber. Cells were illuminated with 500, 800, and 1300 µmol m^−2^ s^−1^ red actinic light for 5 min each in series.

Pulse amplitude modulated (PAM) chlorophyll fluorescence measurements were conducted using a DUAL-PAM-100 measuring device (Oxylab, Heinz Walz GmbH, Germany). *F*_0_ was determined using 1.5 ml aliquots of either N-deprived or N replete cells collected from time course experiments and incubated in the measuring chamber in the dark for 15 min prior to analysis. *F*_m_ is the maximum chlorophyll fluorescence from dark-adapted cells and was determined by application of a 0.6 s saturating pulse of 1870 µmol photons·m^−2^ s^−1^. *F*_t_ was determined after the cells had been illuminated with an actinic light source of 95 µmol photons·m^−2^ s^−1^ for 230 s, during which saturating pulses were applied every 20 s to analyze *F*′_m_ values, where *F*′_m_ is the maximum chlorophyll fluorescence under actinic light [29]. *F*_v_/*F*_m_ as an indicator of the maximum quantum efficiency of PSII was calculated using *F*_0_ and *F*_m_ values according to the formula (*F*_m_ − *F*_0_)/*F*_m_. For calculating the efficiency of PSII photochemistry in illuminated cells (ФPSII = (*F*′_m_ − *F*_t_)/*F*′_m_), *F*ʹ_m_ and *F*_t_ values observed after 210 s of applying actinic light were used [30]. Data were analyzed using SigmaPlot 11 software.

#### 77K fluorescence spectroscopy

The algae cells at a chlorophyll concentration of ∼3 μg 10^−6^ cells were shock frozen in liquid nitrogen and were excited with a broad light source (450–800 nm) produced with a halogen lamp and Schott BG18, Corning 9782, and LOT heat mirror filters. Triplicate emission spectra per sample were averaged and corrected using an internal dye standard. To compare relative fluorescence changes between PSII and PSI maximum absorbance at 685 and 715 nm, respectively, the spectra were normalized to chlorophyll a concentration.

#### Flash-spectrophotometric assays

The levels of P_700_ for wild-type and mutant cells during nitrogen starvation were probed by observing absorbance changes at 700 nm using an instrument described previously [31, 32]. Saturating single-turnover actinic flashes were provided by a xenon flash lamp (approximately 2 J total energy output per pulse, 5 μs duration), filtered with two layers of a red filter (Schott RG665, Schott Glass Technologies, Duryea, PA, USA). Measurements were carried out on dark-adapted cells at room temperature for 10 min. The total amount of photo-oxidizable P_700_ was determined by measuring the 700 nm absorbance change upon illumination in the presence of 100 µM DCMU [(3,4-dichlorophenyl)-1,1-dimethylurea] as artificial inhibitor.

### Acetate quantification

Acetate remaining in culture media was quantified using the Acetate Colorimetric Assay Kit (BioVision, Milpitas, CA, USA). Both N replete and N-depleted TAP medium for wild-type and *tab2* mutant cells contained 17.5 mM acetate prior to culturing at 0 h. One milliliter of the culture was withdrawn from the flask at each time point. The supernatant was filtered through a 0.45 μm filter membrane after centrifugation at 14,000×*g* for 5 min and then was stored at −20 °C. All samples were analyzed using 96-well flat bottom plates (Costar, Corning, NY, USA) following the kit protocol by measuring the absorption maximum at 450 nm in a SPECTRAmax PLUS microplate spectrophotometer system (Sunnyvale, CA, USA).

### Determination of ATP:ADP and NADPH:NADP ratios

ADP was measured using a colorimetric assay kit including a commercial enzyme solution to convert ADP to ATP and pyruvate. The generated pyruvate was quantified spectrophotometrically at 570 nm (BioVision, Milpitas, CA, USA). However, ATP was measured using a colorimetric method based on glycerol kinase activity that utilizes ATP for the phosphorylation of glycerol to generate glycerol phosphate, which is a measurable at 570 nm (BioVision, Milpitas, CA, USA). For both ATP and ADP assays, 1 ml of Chlamydomonas culture was harvested by centrifugation at 14,000×*g* for 2 min at 4 °C. The pellet was flash frozen in liquid N_2_ and disrupted by a TissueLyser II for 30 s. Then, the cells were homogenized in 100 µl of ADP or ATP assay buffer before to additional disruption by sonication in an ice water bath for 5 min. The solution was centrifuged 15,000×*g* for 2 min at 4 °C to remove insoluble material. Four microliters of the supernatant were used to perform the ADP and ATP measurement according to the manufacturer’s protocol (BioVision). The ADP and ATP concentrations were calculated using standard curves. The amounts of NADP^+^ and NADPH were determined using a NADP/NADPH Quantitation Kit according to the manufacturer’s instructions (Sigma-Aldrich, St. Louis, MO, USA). The cells were sonicated in 200 µl NADP/NADPH extraction buffer for 20 min in an ice water bath and then centrifuged 13,000×*g* for 10 min at 4 °C. The supernatant was filtered using a 10 kDa cutoff spin filter (BioVision) to remove proteins from the extract before use in the assay. All measurements were performed in triplicate in 96-well plates and quantified at 450 nm using the colorimetric assay according to the kit instructions.

## Results

### Effects of nitrogen depletion on transcript and protein levels of TAB2 in WT and the *tab2* mutant

**Fig. 1:**
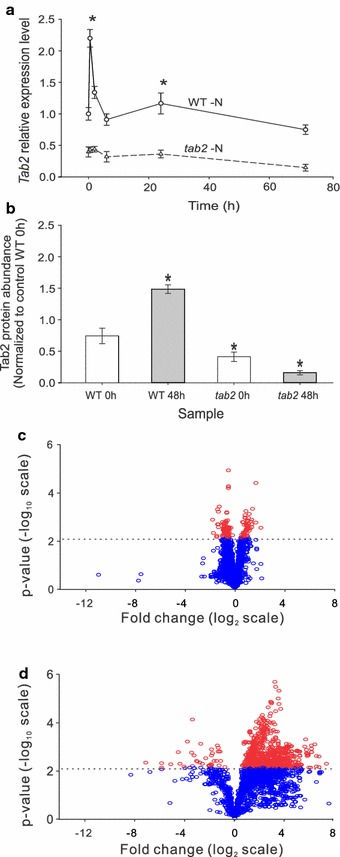
Characterization of Tab2 levels in wild type (CC125) and the *tab2* mutant during the N deprivation time course. **a** Relative expression level of the *TAB2* gene, normalized to the expression of *Actin* (see Additional file 1: Table S1) and then the relative abundance of each time point was normalized to time 0. **b** Abundance of the TAB2 protein, normalized to WT at time 0. Data are presented as mean ± SE (n = 3). *p < 0.05. **c**, **d** Volcano plots of overall proteomic changes in WT and the *tab2* mutant, respectively, comparing fold change of proteins in nitrogen depleted (48 h) relative to nitrogen replete (time 0) conditions. Proteins with significant changes (*p* < 0.05) are shown by *red ellipses*. The stringent cutoff of *p* < 0.01 is shown by a *dotted line*

To investigate the function of TAB2 in *Chlamydomonas* during N deprivation, a comparison between the parental strain (CC-125) and the *tab2* mutant was performed using growth time courses either in the presence (N replete) or absence (N depleted) of nitrogen added to the growth medium (TAP media was used except where indicated, as described in “Methods”). Transcriptome, proteome, and metabolome data were collected across replicate time courses (at least 3 replicate samples per time point were analyzed). We first tested whether the *tab2* mutation affects the expression level of the *TAB2* gene during N deprivation. As shown in Fig. 1a, quantitative real-time PCR demonstrated that the transcript level of *TAB2* in the WT was up-regulated 2.5-fold within the first 30 min following N depletion, whereas in the *tab2* mutant, the *TAB2* transcript levels were 50% of WT at time 0 and remained low throughout the time course. *TAB2* primers for real-time PCR analysis we designed to be specific to *TAB2* gene and not also to *TAB1* which is another (PSI) translation initiation factor of PsaB. The proteomic results revealed that after 48 h of N deprivation TAB2 protein abundance was twofold higher in the WT relative to the time 0 control, whereas in the *tab2* mutant, the TAB2 protein levels were initially significantly lower than in the WT and then decreased more than 61% by 48 h (Fig. 1b). To visualize more global patterns of protein level changes, volcano plots of the full set of identified proteins for both the WT and *tab2* strains were generated, comparing results from 48 h to time 0 (Fig. 1c, d). For the significantly changing proteins in the WT, although more proteins were down-regulated than up-regulated, the fold-changes observed were relatively small (almost all were less than fourfold different in expression level, Fig. 1c). In contrast, for the *tab2* mutant, most proteins that changed significantly in expression increased, and many of these changed by more than fourfold (Fig. 1d). Thus, although the TAB2 protein was originally described as a translation initiation factor for psaB, when it is mutated, significant global changes occur in the proteome. The experiments described below were designed to identify the mechanism responsible for these changes and to determine what affect they had on overall cellular physiology.

### The *tab2* mutant loses viability and has a reduced cell size following N deprivation

**Fig. 2:**
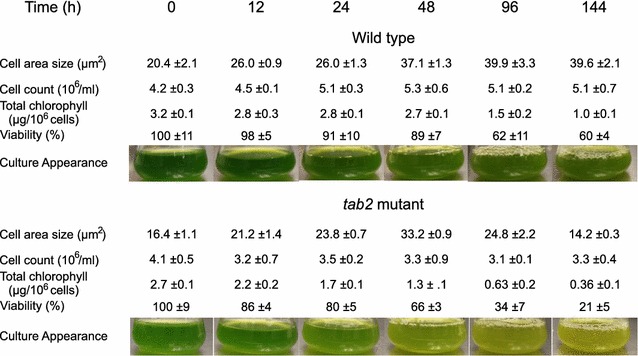
Physiological characterization of the CC125 (wild-type) and *tab2* (mutant) strains cultured in TAP-N medium. All values are averages of three replicates, ±SE

To investigate whether the reduced TAB2 protein level in the mutant cells altered cell physiology during N deprivation, the chlorophyll content, cell viability and cell size were measured across the time courses. Under N deprivation, chlorophyll levels dropped over time in both the WT and the *tab2* mutant, but this reduction (see Fig. 2) was greater in the mutant (80%) than in WT (60% reduction). That corresponded with the observed greater down-regulation of most enzymes involved in chlorophyll and carotenoid biosynthesis in *tab2* compared to WT (Additional file 2: Figure S1). The decrease in chlorophyll content mirrored a dramatic decline of viability in *tab2* cells after 6 days of N deprivation (Fig. 2). Furthermore, the decrease of TAG accumulation in *tab2* under N deprivation was correlated (*R* ^2^ = 0.88) with a decrease in cell cross-sectional area (Additional file 3: Figure S2). However, there was no correlation between carbohydrate levels and cell size, indicating that the increased cell size in the WT corresponded to an increase of TAG content during N deprivation, as has been previously suggested [33].

### Effect on photosynthetic activity and respiration following N deprivation

**Fig. 3:**
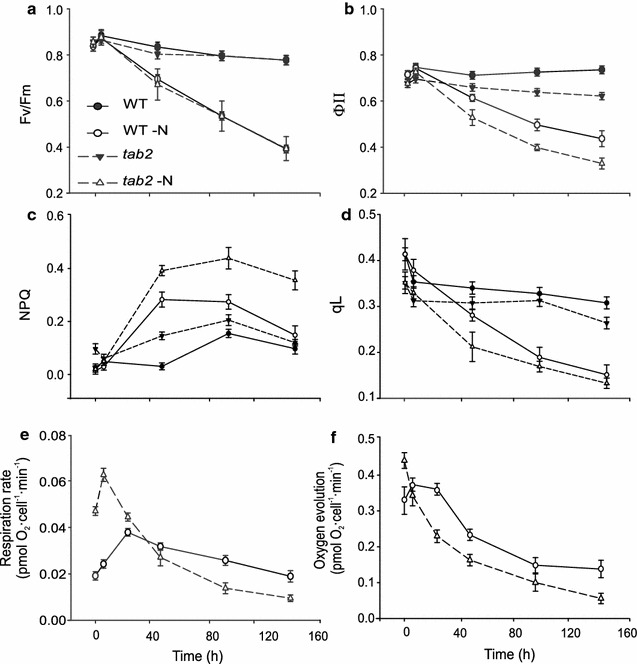
Comparison of photosynthetic properties in WT and tab2 cells. **a** Maximum quantum yield of PSII (*F*v/*F*m) determined in dark-adapted cells grown under N replete (WT *solid circle*, tab2 *solid triangle*) and N-depleted (WT *open circle*, tab2 *open triangle*) conditions. **b** Efficiency of PSII photochemistry in illuminated cells (ФII) under the conditions described above (more details are in “Methods”). **c** Non-photochemical quenching and **d** relative amount of oxidized QA determined in cells grown under N replete and N-depleted conditions. **e**, **f** The rates of oxygen uptake (respiration rate) and evolution were determined in WT and tab2 cells under N-depleted conditions (see legend above of N-depleted condition). Three biological replicates were included in the experiments, with *error bars* representing SE of the mean

To assess whether the observed differences in physiological parameters between WT and *tab2* were related to changes in the photosynthetic apparatus during N deprivation, we used PAM chlorophyll fluorescence to compare changes in the overall photosynthetic capacity in WT and *tab2* cells during the growth time courses. The maximum quantum efficiency of PSII (*F*_V_/*F*_m_) remained constant (around 0.8) in dark-adapted cells growing in N replete conditions, while it steadily decreased in both strains under N-depleted conditions, reaching 0.43 (Fig. 3a), indicative of a decrease in the amount of active photosystem II. No significant differences were observed between the *tab2* and WT strains under these conditions. The efficiency of PSII in light-adapted cells (ФII), on the other hand, decreased 10–20% in the mutant compared to the WT during both N replete and N-depleted conditions (Fig. 3b). Non-photochemical quenching (NPQ) under both N replete and deprivation conditions was also measured (Fig. 3c), showing that NPQ increased markedly in *tab2* following N deprivation and its level was 1.6-fold higher in *tab2* than WT after 4 days. Under N replete conditions, NPQ levels were also higher in *tab2* (4.7-fold) compared to WT by 48 h, suggesting a reduction of LEF capacity in *tab2* relative to WT under both conditions. The efficiency of electron transfer in PSII is partly dependent on the number of open/oxidized quinone A (QA) molecules available. The qL parameter can be used to determine the percent of open PSII reaction centers (oxidized QA pool) [34]. During N deprivation, qL fell more dramatically in *tab2* than in WT, indicating a clear decrease in the abundance of open reaction centers in the mutant as compared to WT, while in N replete conditions, qL levels remained constant throughout the time course in both mutant and WT (Fig. 3d). However, qL levels were lower in *tab2*, indicating a decrease in the abundance of open reaction centers in the mutant. This decrease in photosynthetic efficiency was accompanied by a significant down-regulation of the levels of PSII, PSI and cytochrome *b6f* complex subunits in the mutant (Additional file 4: Figure S3) and demonstrated that the QA pool was increasingly reduced over time in the mutant as compared to the WT.

In addition, the levels of the mitochondrial electron transport chain proteins were observed to be very highly up-regulated in *tab2* compared to the WT during N deprivation (Additional file 5: Figure S4). For this reason, we performed oxygen evolution analysis on both strains during N deprivation. The respiration rate in WT increased initially to 0.37 pmol O_2_ cell^−1^ min^−1^, reaching a maximum at ~24 h (Fig. 3e). The dark respiration rate of the *tab2* mutant increased significantly within 6 h compared to WT (~threefold increase) during N deprivation, but then dropped to approximately half the level of WT during the last few days of the time course. In contrast, the oxygen evolution in illuminated samples in *tab2* decreased to roughly half that of WT by 24 h and remained lower than the WT throughout the rest of the time course (Fig. 3f). Photosynthetic oxygen evolution did not drop in the WT until after 24 h. This delay in establishing hypoxic conditions in the WT compared to the *tab2* mutant is probably due to a prolonged PSII activity in the WT. Taken together, these findings demonstrated that net oxygen production dropped steeply and was accompanied with an increase of respiration rate during the first 24 h in the mutant compared to the WT, suggesting that probably a high degradation of chloroplast in the PSI defective mutant for buildup mitochondrial components, consistent with [7] study on adaptation of Chlamy cells to N deprivation.

**Fig. 4:**
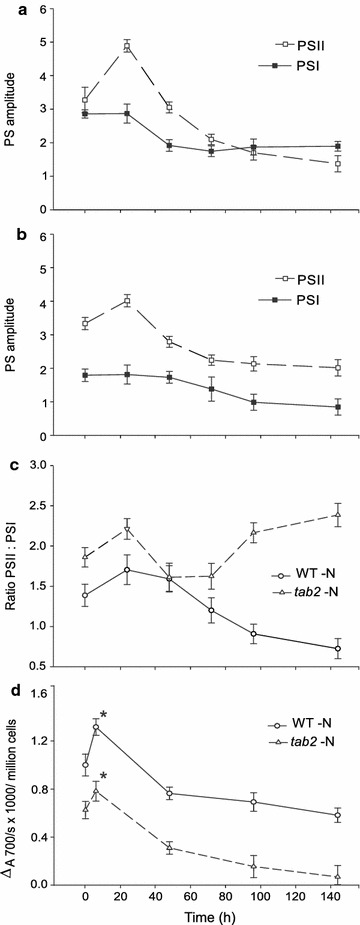
Measurement of the relative PSII and PSI amplitudes by 77K fluorescence spectroscopy. **a** The amplitude of PSII and PSI in WT during N deprivation. **b** The amplitude of PSII and PSI in *tab2* during N deprivation. **c** The PSII/PSI ratio for WT and *tab2* cells during the N deprivation time course. **d** Spectroscopic quantification of P_700_^+^ content under N-depleted conditions in WT and *tab2* cells. Data were collected after illumination in the presence of DCMU

To gain further insight into the effects of the *tab2* mutation on the amount of intact PSII and PSI complexes during N deprivation, we used 77K fluorescence analysis. The amplitude of the PSII-associated signal (around 685 nm) in WT increased within the first 24 h, after which it dropped through the rest of the time course. In contrast, the PSI-associated signal (around 715 nm) in WT cells did not increase at first, but decreased after 24 h and then remained constant across the rest of the time course (Fig. 4a; Additional file 6: Figure S5). In *tab2* cells, the response was very similar but less pronounced for PSII (Fig. 4b). For the PSI-associated signal, the time course for *tab2* is similar to WT but the absolute amplitude is about 50% lower, indicating reduced abundance of PSI complexes as expected for a PSI translation initiation mutant (see also next paragraph). Consequently, the ratio of PSII to PSI was significantly higher in the *tab2* mutant relative to the WT (Fig. 4c), supporting the proteomics and transcriptomics results described above.

To verify whether the *tab2* mutation resulted in a general impairment of functional PSI during N deprivation, we measured the absolute amounts of photo-oxidizable P_700_ (Fig. 4d) in the presence of the PSII inhibitor, 3-(3,4-dichlorophenyl)-1,1-dimethylurea (DCMU, Diuron). The level of intact PSI centers in the mutant was initially about 60% of the WT. This is in line with 77K fluorescence results (see above). During the N deprivation time course, the PSI content in the WT and *tab2* peaked within the first 6 h and then decreased by about 15% in the WT and 50% in the mutant by 48 h. By 4 days of N deprivation, the amount of PSI had decreased only slightly in the WT, while in the mutant, PSI had reached very low levels. These findings confirm the important role of the TAB2 protein in maintaining the levels of PSI and therefore retention of active photosynthetic apparatus during N deprivation. These results are also consistent with a previous suggestion [14] that the PSI reaction center proteins are more stable than PSII during N deprivation in microalgae, although that had been not reported for *Chlamydomonas* until now.

Although the chlorophyll content decreased by 12 and 22% in the WT and the mutant after 12 h of N deprivation, respectively, functional fluorescence measures, *F*v/*F*m and Φ_ii_, decreased only slightly, suggesting that those decreases in chlorophyll content had only a minimal impact on the efficiency of PSII. The observed decrease in PSI subunits and the measurable oxidizable P_700_ levels in the *tab2* mutant (threefold decrease compared to the WT) led to the question of whether that decrease in PSI levels might have an impact on NADPH levels and therefore an impact on TAG synthesis in the mutant. We hypothesized that the TAB2 protein, by playing a crucial role in maintaining active PSI complex, may be indirectly responsible for enabling the production of TAG in the WT during N deprivation, at least within the first 24 h following N deprivation, and that in the *tab2* mutant, reduced levels of PSI could have broad reaching impacts on metabolism.

### The *tab2* mutation leads to drastic changes in ATP/NADPH ratios during N deprivation

**Fig. 5:**
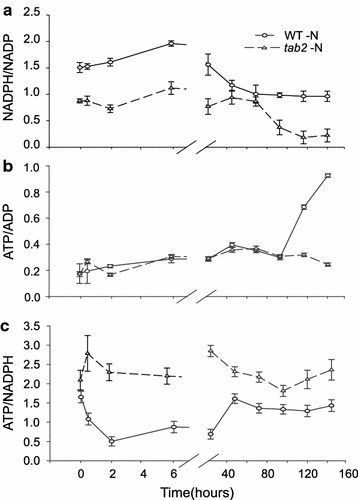
Changes in the NADPH, NADP^+^, ATP, and ADP content relative to each other in WT and *tab2* cells across the N deprivation time course. **a** Ratio of NADPH to NADP^+^; **b** ATP to ADP; and **c** ATP to NADPH. All values are averages of three biological replicates with *error bars* indicating SE

To further understand the function of TAB2 in modulating the relationship between photosynthesis and TAG accumulation that was observed in this investigation, we monitored the change in ATP, ADP, NADPH, and NADP^+^ levels in cells across the growth time courses and compared these values to each other. In the very early phase of N deprivation, by 2 h, the NADPH/NADP^+^ ratio increased significantly in the WT, while it dropped in the *tab2* cells. By 24 h, the WT ratio dropped and then it remained consistently around 1 throughout the rest of the time course (Fig. 5a). On the other hand, the *tab2* cells recovered quickly, with the ratio returning to normal (around 1) by 6 h. By 48 h, however, the *tab2* ratio had dropped significantly and it remained low throughout the rest of the time course compared to the ratio in WT. In contrast, the ATP/ADP remained consistently low for the WT but slowly increased in the *tab2* mutant over the initial few days of the time course (Fig. 5b). The increase in ATP in the WT during the last stages of N deprivation is likely the result of cyclic electron flow around PSI, which decreases the flow of electrons into the NADPH pool. That implies that there is an increase in demand for photosynthetically generated ATP relative to NADPH, most likely needed for maintenance and remodeling of the Chlamydomonas proteome during N deprivation [8].

The ATP/NADPH ratio thus declined from 1.5 to 0.6 in WT cells during the first 6 h of N deprivation (Fig. 5c). This ratio remained low during the first 24 h, but returned to ca. 1.5 by 48 h, where it remained for the duration of the time course. In contrast, the initial decline was absent in the *tab2* mutant, and the ATP/NADPH ratio remained relatively constant for the duration of the time course, with a value of about 2–2.5. This difference in the ATP/NADPH ratio response for the *tab2* mutant versus the wild type may be the result of a difference in demand for NADPH associated with FA biosynthesis in *tab2*. Therefore, experiments to test this hypothesis were performed, as outlined below.

### The *tab2* mutation leads to an increase of TAG and starch levels under N replete conditions

**Fig. 6:**
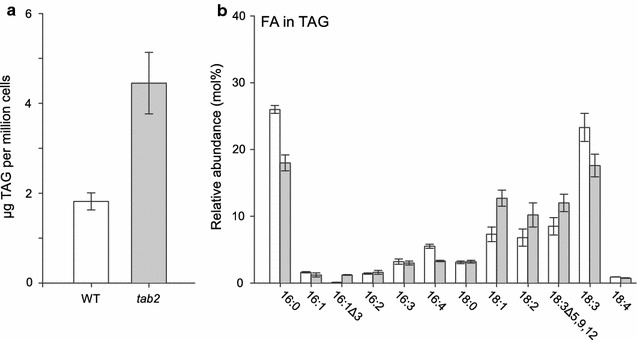
Analysis of lipids in WT and *tab2* mutant cells grown in N replete medium. **a** Comparison of TAG content in the WT versus *tab2* cells. **b** Mol (%) of specific fatty acids in TAG isolated from WT (*white*) and *tab2* (*gray*) cells. Values are averages of triplicate biological samples. *Error bars* indicate SE of the mean

To determine what changes in metabolism resulted from the *tab2* mutation, the levels of primary metabolites and neutral lipids and FA composition were compared across the time courses, focusing first on the N replete time course, where cells were grown in TAP media for 8 days until nutrients ran out. TAG amounts in the mutant were found to be greater than twofold higher than in the WT across the first 24 h of the N replete time course (see Fig. 6a). The *tab2* mutant had a higher starting level of neutral lipid content but both the mutant and WT significantly increased neutral lipid levels per cell by the same magnitude within 48 h. Neutral lipid levels in *tab2* then decreased to levels just higher than WT and these levels remained relatively constant in both cell lines for the remainder of the time course, after 8 days (Additional file 7: Figure S6A). Thus, under the initial N replete conditions, the higher overall metabolic capacity of the WT cells not defective in PSI led eventually to the same TAG accumulation end point as *tab2* in the long time course when the media progressively ran out of N and then C.

Compared to the WT, the *tab2* mutant had higher levels of 18:1 (12.7 versus 7.3%) and 18:2 (10.2 versus 6.8%) fatty acids (Fig. 6b). In contrast, WT cells were rich (compared to the mutant) in polyunsaturated FA (PUFA) species containing 3 and 4 double bonds, characteristic of membrane lipids, such as 16:4 (5.5% in WT versus 3.3% in *tab2*) and 18:3 (23.3 versus 17.6%) (Fig. 6b). The knockdown of *tab2* levels leads to an increase in specific enzymes required for elimination of reactive oxygen species. Oleate (18:1) is a major constituent of wild-type TAG, accounting for 25% of the acyl groups in TAG, but represents only up to 10% of acyl groups in diacylglyceryltrimethylhomoserine and phosphatidylethanolamine [28], major lipid classes in Chlamydomonas membranes. Thus, a high relative 18:1 level in the mutant growing under N replete condition is indicative of an emphasis by the cells on TAG production instead of cell membrane production at the end of the time course (Additional file 7: Figure S6B, C).

**Fig. 7:**
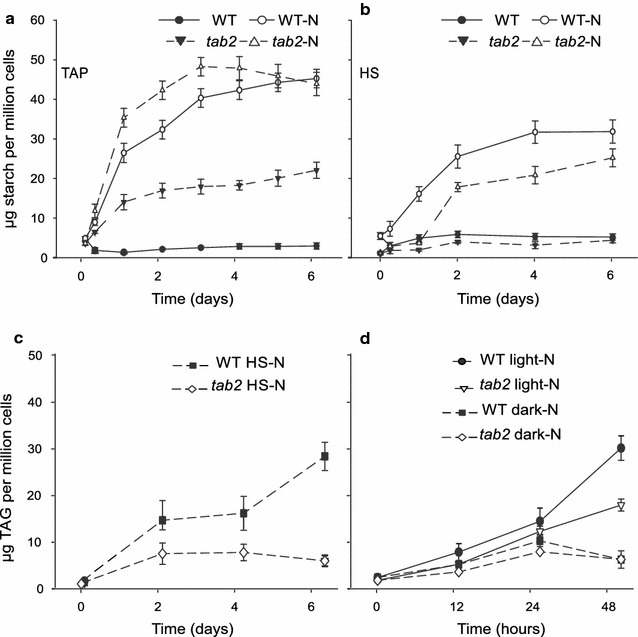
Changes in TAG and starch content in WT and *tab2* cells in growth time courses under different growth conditions. **a** Time courses of starch accumulation in cells grown in TAP medium or **b** HS medium under N replete and N-deprived (–N) conditions. **c** TAG levels in cells growing in HS medium lacking N. **d** TAG levels in cells that were precultured in TAP medium and then resuspendend in either TAP and TAP-N medium at 4 × 106 cells ml^−1^ and grown for 2 days in the dark or light. Values in *all plots* indicate the mean of three independent experiments (±SE)

To better understand the partitioning of carbon reserves in *tab2*, and to see what role starch synthesis may play in that process, we monitored the starch levels in *tab2* and WT cells during the N replete time course. Starch levels in the mutant rose rapidly during the first 24 h and reached levels 9 times higher than was observed for the WT by the end of the time course (Fig. 7a). This enhancement of starch and TAG production in the *tab2* mutant under N+ conditions, particularly in the presence of acetate, leads to the suggestion that altered photosynthetic efficiency (due to reduced PSI levels) is a condition required to promote energy storage compound accumulation without N depletion. This will be described in more detail below.

### The role of acetate assimilation in TAG and starch production in the *tab2* mutant

Under optimal growth conditions, WT cells do not accumulate high levels of energy storage compounds (Additional file 7: Figure S6; Fig. 7a). When algae encounter stresses, such as a lack of nitrogen, cell division slows down and carbon is remobilized to produce energy storage products, such as lipids and/or starch in response to altered nutrient availability. In our study, the TAG and starch content in the *tab2* mutant increased significantly under mixotrophic conditions (where acetate was present as a carbon source) not lacking for N (Fig. 6a), something that did not occur in the WT cells. Thus, the external carbon source may play a significant role in production of energy storage products in the *tab2* mutant. To verify this hypothesis, we monitored acetate utilization in *tab2* and WT cells across the N replete and N deprivation time courses. The acetate uptake in N replete media by *tab2* cells was ~2.3-fold higher than by WT cells after 24 h (Fig. 9), which is also in line with the higher respiration rate observed in the mutant compared to the WT (Fig. 3e). Coincidently, the time point at which lipids and starch began to increase in the mutant under these conditions was just when the levels of acetate in the media began to drop. To further confirm the high use of acetate caused by the *tab2* mutation, algal cells were grown in HS medium (lacking acetate) or in the dark. The TAG content of the cells showed no increase under these conditions compared to those grown under the mixotrophic conditions (Fig. 7c, d). Moreover, the starch content remained lower than that observed in the WT (Fig. 7b). In addition, acetate uptake was higher in the mutant than in the WT within the first 24 h following N depletion, enabling the observed starch accumulation in the mutant (Fig. 7a). This higher rate of acetate assimilation in *tab2* cells under N deprivation can be explained by the observed up-regulation of the two enzymes, ACK2 (A8IR49) and PAT2 (A8IZZ9), involved in acetate uptake via the acetate-phosphate pathway (Additional file 11: Figure S10A).

**Fig. 8:**
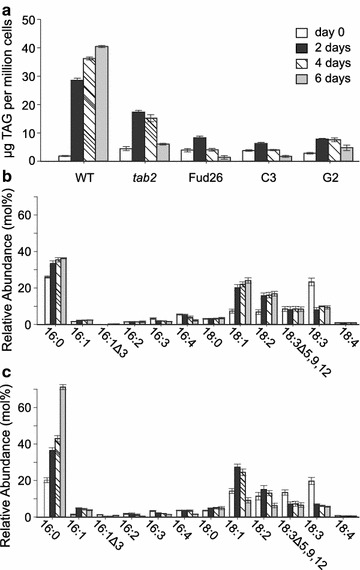
Detailed lipid analysis of wild-type and *tab2* mutant cells grown in N-depleted medium. **a** Changes in TAG content over the time course in the wild-type, *tab2* mutant, two strains detective in *PsaB* gene function (Fud26 and G2) and one strain defective in *PsaA* (C3). **b**, **c** Fatty acid composition in TAG isolated from the wild-type and the *tab2* mutant, respectively. Values are averages of triplicate biological samples, ±SE

**Fig. 9:**
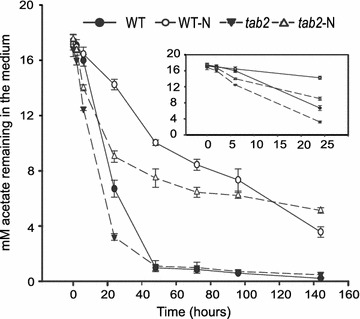
Acetate consumption in WT and *tab2* cells grown in TAP medium under N replete or N-depleted (–N) conditions, measured by reduction of acetate concentration in the medium. The starting acetate concentration for both conditions was 17 mM. Values are averages of triplicate biological samples. *Error bars* indicate SE

The above results demonstrated lower starch and lipid accumulation in the *tab2* mutant for cells grown in photoautotrophic or heterotrophic culture conditions compared to mixotrophic conditions with either N present or absent, with a concomitant increase in acetate utilization (Figs. 7, 8, 9). Thus, the simultaneous presence of acetate and light appears to be a necessary condition for the small increase of TAG in the *tab2* mutant, otherwise the mutant accumulates preferentially starch over lipids compared to the WT, due presumably to the lower energy requirement for the biosynthesis of the former over the latter [35]. These results are consistent with the idea that the addition of an external carbon source can quantitatively substitute for photosynthetic carbon assimilation to drive growth and storage compound accumulation in *Chlamydomonas* under some circumstances (i.e., defective PSI). Here, it is noteworthy to mention that the experiments reported above were possible to be performed since we have been able to grow in this present work the mutant in liquid culture with a delay of 4 days to raise the exponential phase when its cultured in TAP medium and a delay of 8–10 days when its cultured in HS medium as compared to the WT growth rate. However, we have observed the same growth rate as Dauvillee et al. [20] reported that mutant cells were unable to grow photoautotrophically, and *tab2* grew poorly on acetate-containing medium under high light (60 µE/m2/s) in agar plates. The delay in growth of *tab2* in HS medium could suggest the development of a suppressor mutation; however, it is unlikely that suppressor mutation promoted the growth only in liquid medium and not in plates. Furthermore, we have been not able to detect any suppressor colonies after the mutant cells growing in liquid HS medium were spread in HS agar plate and allowed to grow for two weeks, because of that we have been working only with HS liquid seed culture to conduct our experiments.

### Partitioning of carbon into TAG and starch following N deprivation is altered in the *tab2* mutant

TAG levels were found to increase in the WT only after 48 h of N deprivation (Fig. 8a). In contrast, the *tab2* mutant produced 40% less TAG compared to the WT under these conditions by 48 h, and at 6 days possessed only 17% of the TAG that the WT accumulated (Fig. 8a). Other mutant strains that were defective in PsaB and PsaA also accumulated low amounts of TAG during N deprivation (Fig. 8a). This suggests that changes in lipid profiles in the *tab2* mutant belong at least partially to a generic cellular response to defects in PSI. Under N deprivation, WT cells demonstrated a major change in the fatty acid profile of the purified TAG compared to cells grown in N replete conditions. In particular, there was a progressive decrease in omega-3 PUFAs (18:3, 16:3, 16:4) and a corresponding increase in 16:0, 18:1, and 18:2 [36] (Fig. 8b). However, after the initial increase as in wild type, the 18:1 and 18:2 levels in *tab2* cells started to decrease after 2 days of N deprivation, leading eventually (at 6 days) to a threefold decrease compared day 2. It is worthwhile noting that the highest increase for any FA in the mutant was for palmitic acid (fourfold increase) after 6 days of N deprivation (Fig. 8c). The dramatic decrease of acyl-ACP desaturase (A8IQB8), delta-9-ACP desaturase (A8IUT7), and palmitoyl-monogalactosyldiacylglycerol Δ7-desaturase (FAD5, A8JEN2) levels (Additional file 8: Figure S7) may explain this increase in 16:0. Likewise, the abundance of transcripts encoding enzymes in fatty acid and lipid metabolism (Additional file 9: Figure S8), as well as their protein levels, were either unchanged or decreased in *tab2* versus WT during the N deprivation time course (Additional file 8: Figure S7). The expression profiles of the diacylglycerol acyltransferases (DGATs) and diacylglycerol acyltransferase type-two enzymes (DGTTs) responsible for the final step of TAG biosynthesis suggested that they were highly down-regulated (in general) in *tab2* versus WT during the course of the N deprivation experiment (Additional file 9: Figure S8). However, the transcript levels for specific isoforms, DGAT1, DGTT2, and DGTT5, increased initially after the onset of N starvation (Additional file 9: Figure S8). The early increase of DGAT1 and DGTT2 expression agrees with a previous study of the response of Chlamydomonas to nitrogen deprivation [36], whereas DGTT5 expression has not been detected under either N or iron starvation [36, 37]. In this study, the abundance of DGTT5 transcripts increased early and peaked by 0.5 h at about twofold higher levels in the mutant versus the WT, after which it decreased (Additional file 9: Figure S8). In addition, the levels of enoyl-CoA hydratase (A8JBL6), involved in the second step of β-oxidation of fatty acids, decreased twofold in the mutant compared to the WT after 2 days of N deprivation (Additional file 8: Figure S7). The reduction of β-oxidation was demonstrated to accompany an increase in neutral lipid levels in *Thalassiosira pseudonana* [38]. Thus, the changes in TAG composition observed in the mutant compared to the WT appear to be the result of significant changes in the expression and activity of a number of enzymes related to both TAG biosynthesis and FA utilization, including the activity of specific DGATs and DGTTs. This points to a potential critical role for these enzymes, while perhaps not in regulating overall flux into or final levels of TAG, in determining the FA composition of the TAG that accumulates, and thereby at least partially modulating reorganization of membranes as the cell transitions from a growth to a quiescent state [39, 40].

In contrast to the low accumulation of TAG, starch levels were significantly higher in *tab2* than in the WT during the time course of N deprivation (Fig. 7a). The expression levels of many genes encoding different classes of enzymes involved in starch biosynthesis generally increased under N deprivation in *tab2* compared to the WT (Additional file 9: Figure S8). In particular, the transcript levels for certain isoforms of these enzymes, such as glucose-6-phosphate isomerase 1 (PGI1), starch branching enzyme isoforms 1 and 2 (SBE1 and SBE2) and soluble starch synthase isoform 3 (SSS3), increased rapidly in the mutant upon N depletion, following a pattern similar to that seen for DGAT and DGGTs, and peaked between 0.5 and 2 h before decreasing (Additional file 9: Figure S8). However, other isoforms of some of these enzymes, such as SBE3 and SSS2, were expressed at higher levels in the WT, and others, such as SSS1, SSS4, and SSS5 remained elevated in expression for a longer time in the mutant. Thus, it was difficult to determine how any given enzyme isoform contributed to the pattern of starch accumulation. In any case, it was clear from the proteomics data (Additional file 10: Figure S9) that by 2 days post-depletion, the mutant had returned to a state of significantly reduced expression for almost all enzymes involved in starch biosynthesis and elevated accumulation of proteins involved in starch degradation, relative to the wild type, in apparent preparation for the subsequent decrease in starch levels that was observed shortly thereafter (Fig. 7a). This enhancement of starch production in the *tab2* mutant versus TAG production under N-depleted conditions can be explained by the data presented above regarding ATP, NADPH, and photosystem levels. Because the *tab2* mutant cells are unable to maintain an equilibrated energy balance with adequate ATP and NADPH production levels, the carbon that they fix cannot be efficiently converted to TAG, which is a more energy and reducing potential demanding storage molecule than starch. Determining how (and if) post-translational regulation plays a role in regulating starch production in the *tab2* mutant versus the WT was beyond the scope of this project and will be the subject of future research.

### Omics analysis validates the effect of the *tab2* mutation on carbon partitioning during N deprivation

If the partitioning of carbon into TAG and starch was altered in the *tab2* mutant, then it was likely a result of reprogramming of the central carbon metabolic network. To test this hypothesis, all proteins and metabolites within central metabolism were evaluated for their abundance levels across in both wild-type and mutant cells over a developmental time courses.

#### The glyoxylate cycle is down-regulated and the TCA cycle up-regulated in the *tab2* mutant

A decrease in the protein abundance of the isocitrate lyase (ICL1) and malate synthase (MS1) isoforms involved in the glyoxylate cycle was observed after 2 days following N depletion (Additional file 11: Figure S10B) in the mutant relative to wild type. This decrease was coincident with a high increase in TCA enzymes after 2 days of N deprivation. Notably, the levels of the mitochondrial citrate synthase (CIS1) and the four succinate dehydrogenases (SDH1, SDH2, SDH3, and SDH4) that catalyze the oxidation of succinate to fumarate increased up to threefold (Additional file 11: Figure S10B). These results suggest significant enhancement of TCA cycle activity in the mutant during N deprivation, which is also in line with the higher respiration rate observed in the mutant during the first 48 h compared to the WT (Fig. 3e).

#### Glycolysis, gluconeogenesis, and amino acid metabolism are altered in the *tab2* mutant

The TCA cycle is an amphibolic pathway that functions in central metabolism of plants not only to feed metabolic intermediates to the respiratory system for energy production, but also to produce intermediates that leave the cycle to be converted into sugars and amino acids, among a host of other compounds. The protein levels of two enzymes specific for glycolysis, phosphofructokinase 1 and 2, PFK1 (A8HX70) and PFK2 (A8IYM0), increased (Additional file 10: Figure S9B), while the amounts of phosphoenolpyruvate carboxykinase (PCK1A, A8J0N7) and fructose 1,6-bisphosphatase (FBP1, A8IKQ0), required for gluconeogenesis, were greatly decreased in the mutant compared to WT after 2 days of N deprivation (Additional file 10: Figure S9A). However, the relative expression levels for the two PCK1A isoforms and FBP1 were higher in *tab2* than the WT by 2 h following N deprivation (Additional file 9: Figure S8). Moreover, the increase in glycolytic state in *tab2* relative to the WT after 2 days of N deprivation was also supported by the up-regulation of two alpha-amylases, AMYA1 (A8IYY5) and AMYA2 (A8J4D3), and an increase in glucose levels as well as the sugar phosphate, Glc6P (Additional file 12: Figure S11). A previous report demonstrated that Chlamydomonas cells respond rapidly to N deprivation by immediately turning on a gluconeogenic state and then by about 6 h repatterning metabolism to shift to a largely glycolytic state [41]. These results suggest that such a response was enhanced in the TCA cycle of *tab2* cells.

In addition, amino acid synthesis was reorganized in the *tab2* mutant compared to the WT. A strong decrease of the levels of the enzymes involved in the synthesis of aromatic amino acids, alanine, valine, isoleucine and leucine was observed. In contrast, an increase of the cytosolic glutamine synthetase (GLNA1) and the chloroplastic form (GLNA2), as well as glutamate dehydrogenase (GHD2) and aspartate aminotransferase (AST1) was observed. This was accompanied by a general stimulation of arginine synthesis, as evidenced through the up-regulation of argininosuccinate synthase (AGS1), argininosuccinate lyase (ARG7), and ornithine carbamoyltransferase (OTC1) (Additional file 13: Figure S12). These results led us to carefully examine the levels of each of the amino acids across the time courses. As expected, the amounts of glutamine and arginine showed greater accumulation within the last 3 days of the N deprivation time course in *tab2* compared to the WT (Additional file 14: Figure S13). The increase in accumulation of these amino acids is likely the result of the increase in flux through the TCA cycle to produce substrates for amino acid biosynthesis.

All of these results suggest that a shift towards the synthesis of specific amino acids as opposed to general amino acid degradation occurs in the *tab2* mutant during N deprivation. Glutamine and arginine play a critical role in C/N partitioning and N assimilation in plant cells, and considering the N-depleted conditions experienced by the cells in the experiment, it appears that the mutant cells actively maintain high levels of these metabolites (by rapid turnover) compared to the WT cells, thus enabling a rapid response to changing N levels that the cells may experience. Interestingly, it was also noticed that the amounts of several enzymes involved in autophagy, such as proteases (cysteine protease CEP, serine proteases, Deg-P type protease) are significantly increased in *tab2* (Additional file 15: Figure S14B) and could also contribute to the high level of amino acids detected above.

#### Fatty acid catabolism is increased in the *tab2* mutant during N deprivation

**Fig. 10:**
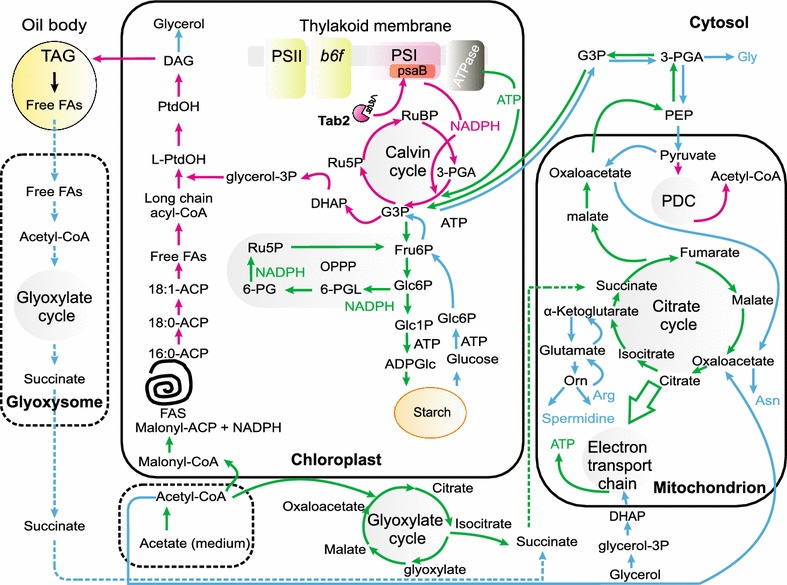
Model of the major metabolic consequences of the *tab2* mutation and how it affects TAG biosynthesis. The specific alterations to metabolic flow at different times during the growth time course are indicated by *colored arrows*. See text for a discussion of how this model is supported by experimental evidence. Processes that are reduced or enhanced, respectively, during the early phase of N deprivation (during the first 2 days) are indicated by *pink* and *green arrows*. Processes that are significantly altered during later stages of N deprivation are indicated by *blue arrows*. Predicted pathways are represented by *dashed lines*. Not all intermediates and reactions involved are shown to make the figure easier to read

The increase in glycerol amounts (Additional file 12: Figure S11) and the level of dihydroxyacetone kinase (DAK1, A8JB14) after 2 days of N deprivation followed the up-regulation of many enzymes involved in fatty acid catabolism (Additional file 8: Figure S7) suggests that an increase in fatty acid degradation may be used by *tab2* cells to feed acetyl-CoA into the TCA cycle to support the reprograming of metabolic flux (Fig. 10).

### The knockdown of TAB2 levels leads to an increase in specific enzymes required for elimination of reactive oxygen species

Any process that disrupts the maintenance of proper ATP/NADPH ratios can also lead to the formation of reactive oxygen species (ROS) [42]. Protein levels of many enzymes involved in the protection against ROS in mitochondria (e.g., MSD1, GPX3, and CCPR1) and in the chloroplast (FSD1, APX1) were found to be strongly up-regulated only in *tab2* (not in WT) during N deprivation (Additional file 15: Figure S14A), although their mRNA transcript levels did not show a significant change compared to the WT (Additional file 9: Figure S8). Several other oxidative stress-related enzymes were also up-regulated in *tab2*, such as GST1 (A8JBB4) and GSH1 (A8IA77), and could function in protecting against singlet oxygen stress [43] by being involved in the detoxification of ROS produced as a result of decreased PSI levels [44]. In contrast, MDAR (A8JDG4), which is required to generate ascorbate using NADPH [45], was up-regulated instead, but only in the WT, which as described above maintained higher NADPH levels than the mutant. The divergence of transcript and protein expression in these cases implicates post-transcriptional regulation of oxidative stress protection. Herein, the loss of the ability to accumulate TAG in *tab2* was correlated to the up-regulation of enzymes involved in ROS protection, supporting the hypothesis that TAG synthesis is not only a consumer process of photosynthetic resources for carbon storage [25], but may also be important for the efficient oxidation of reduced NADPH to prevent over reduction of electron transfer systems and ROS production.

## Discussion

### Altered photosynthetic activity in the presence of acetate and N enhances TAG and starch accumulation in the *tab2* mutant

The *tab2* mutation leads to the accumulation of neutral lipids and starch in cells growing mixotrophically on acetate medium supplied with sufficient N amounts, particularly within the first 2 days of the growth time course (Figs. 6, 7a). This was an unexpected result. Previous studies with other *C. reinhardtii* (particularly WT) strains observed a reduction in photosynthetic efficiency, growth, and carbon fixation in N-depleted cells [16]. One explanation for the higher accumulation of these storage compounds in the *tab2* mutant compared to WT may be increased activity of acetyl-CoA synthetase and the glyoxylate pathway. Acetate consumption was higher in the mutant than the WT under N replete conditions (Fig. 8), thus confirming this hypothesis. However, no significant accumulation of starch and neutral lipids was observed when the cells were grown without acetate or under dark conditions. This led to the hypothesis that altered photosynthetic efficiency, such as was caused by the knockdown of the TAB2 protein levels in this study, may be a condition required to promote energy storage compound accumulation in Chlamydomonas cells, independent of removal of N or other nutrients from the medium, but that sufficient carbon must nevertheless be available. Thus, it is possible that specific alterations of photosynthetic efficiency may be identified that lead to enhanced lipid accumulation in algal cells without the requirement for changes in media conditions that lead to cessation of growth.

In response to N deprivation stress, the accumulation of TAG in the WT lagged behind starch production [25]. That may be due to the temporal separation between the responses of transcript abundances for these two pathways that was observed in the WT strain [10]. However, this temporal separation was affected by the *tab2* mutation under N replete conditions, where neutral lipids accumulated within the first 2 days, at the same time as the accumulation of starch. Lipid levels then decreased while starch increased slightly until day 6 (Figs. 6, 7a). The observation that neutral lipids accumulate at a faster rate than starch in the mutant during the first 2 days under N replete conditions suggests that the accumulation of TAG in the presence of acetate is more likely a direct response to disrupted photosynthetic efficiency than to the prior accumulation of starch, which is consistent with the view that in Chlamydomonas starch and TAG accumulation have different purposes [25]. These findings suggest that TAG accumulates not only as a carbon sink for growth arrested cells but also as a means to protect the photosynthetic machinery under adverse conditions, as suggested by [28], or it could serve as a storage pool that can be readily remobilized to restore thylakoid membranes and photosynthetic activity [8].

### Metabolic shift towards amino acids accumulation in the absence of TAG biosynthesis during N deprivation

The transcript mRNA level of ICL and MAS1 were up-regulated after only 2 h following N deprivation and remind higher up to 24 h in the mutant versus WT (Additional file 9: Figure S8). This increase was followed by the accumulation of malate (Additional file 12: Figure S11) and a higher respiration in the mutant (Fig. 3e) particularly during the first 6 h following N deprivation, indicating that the excess of acetyl-CoA was fueled most likely into glyoxylate cycle after the onset of N deprivation and then oxidized to produce NADH and FADH2 for the respiratory chain that leads to high production of ATP in the mutant (Fig. 10). In line, several genes involved for gluconeogenesis increased during the first hours of N deprivation (Additional file 9: Figure S8) coordinately with the increase level of G3P (Additional file 12: Figure S11) suggesting that the abundant ATP is exported out of the mitochondria and participates to reverse phosphoglycerate kinase activity in the cytosol and leads to the formation of G3P. Since we observed a high accumulation of starch in the mutant as compared to the WT followed by a dramatic decrease of G3P level (Additional file 12: Figure S11), it is possible that a major part of the G3P imported to the chloroplast can be used for the gluconeogenesis and the high accumulation of carbohydrates than the accumulation of fatty acids (Fig. 10), thus supporting our interpretation that TAG synthesis serves as a cul-de-sac for the excess of acetyl-CoA produced from acetate in the presence of an adequate amount of reductant.

After 2 days of N deprivation and with a decrease of acetate assimilation rate, a strong decrease of respiration and in the amounts of enzymes involved in the glyoxylate cycle and gluconeogenesis was observed in the *tab2*. This decrease was correlated a high increase of the amount of TCA enzymes (Additional file 11: Figure S10B). One possibility is raised by this observation that the acetyl-CoA was fueled preferentially to TCA cycle without any bypass to glyoxylate cycle. The up-regulation of TCA cycle enzymes was coupled with the up-regulation of glycolysis enzymes, an accumulation of glycerol (resulted probably from an active fatty acids turnover) and an elevated amount of several amino acids in the *tab2* mutant after 4 days of N deprivation. The increase of glutamate synthesis was concomitant with up-regulation of ARG9 an acetyl-ornithine aminotransferase involved in the recycling of glutamate from ornithine (Additional file 13: Figure S12). Since the amount of alpha-ketoglutarate was remained constant in *tab2* during N deprivation (Additional file 12: Figure S11). This observation suggests that once the glutamate is produced, it is distributed to other amino acids that in turn participate in the regeneration of alpha-ketoglutarate that can then be used to re-fuel the TCA cycle with no net energy expense [17]. Additionally, the synthesis of amino acids can serve as important precursors for metabolite biosynthesis, such as polyamines, nucleotides, and enzyme cofactors. Pursuant to this observation, the amount of the spermidine was noted to have accumulated slightly more in *tab2* than the WT. Elevated amino acids have been also observed in Chlamydomonas after knockout of isocitrate lyase [46]. This suggests that the accumulation of amino acids maintains the mitochondrial pH and reduces the acidic stress caused by the accumulation of organic acids. These results suggest that the knockdown of TAB2 leads to a harmful metabolic shift towards amino acids during N deprivation in the presence of acetate, which can be avoided if the cells maintain the correct energy balance required for the accumulation of TAG.

### A proposed model for regulation of metabolism in the *tab2* mutant

One of the major objectives of this study was to better understand why algal cells produce TAG under nutrient-stressed conditions. In response to N starvation stress, acetate (if provided in the growth medium) is assimilated to form acetyl-CoA and in WT cells is available to feed preferentially to the glyoxylate and citrate cycles during the first hours following the nutrient stress to yield succinate, malate, and oxaloacetate, which in turn can be converted into phosphoenolpyruvate to be used by gluconeogenesis to support cellular growth or other energy demanding metabolic processes, which are also supported by a fully functional photosynthetic apparatus. However, because nitrogen deprivation has been sensed and metabolism repatterning begun [41], the cells initiate a process whereby they switch from that gluconeogenic state to first a glycolytic state and eventually to accumulation of TAG instead of enhanced cellular growth. The quick response at the onset of N deprivation is an energy demanding process that consumes ATP and leads to a dramatic disruption of the ATP/NADPH ratio, as was described in this report. The WT cells, with fully functional PSI, are able to support the cellular growth for a time, during the transition to the strongly glycolytic state. But eventually they cease growth and turn on TAG production, leading eventually to accumulation of significant TAG levels.

In the *tab2* mutant cells, which have not only significantly reduced levels of PSI compared to WT but also dramatically altered proteomes as well, the metabolic response to N deprivation is quite different. During the early phase of N deprivation (during the first 2 days), NADPH production is reduced compared to the WT, as highlighted by the large increase in the ATP:NADPH ratio described above. This leads to reduced Calvin cycle activity, reduced fatty acid biosynthesis and lower TAG production (the pink arrows in Fig. 10 indicate metabolic processes that are reduced in the *tab2* mutant during this early stage). During the first 2 days, acetate imported from the medium could theoretically be used to generate malonyl-CoA and thus support FA biosynthesis, but because of the reduced NADPH availability (due to lower PSI levels), FA and TAG syntheses are not well supported. Instead, the excess acetyl-CoA (formed from the acetate imported from the medium) feeds into the glyoxylate and citrate cycles and leads to elevated production of ATP and production of glyceraldehyde-3-phosphate (green arrows in Fig. 10), as was seen for the WT, to be used to support elevated gluconeogenesis. However, instead of being used to support cell growth, the oxidative pentose phosphate pathway (OPPP) is activated to produce what NADPH is required for maintenance of cellular function, and gluconeogenesis serves the function of producing starch, a lower reductant-demanding storage molecule than TAG.

During the later stages of N deprivation, when acetate has been depleted from the medium, protein synthesis/regeneration cannot be supported due also to reduced N availability. Amino acid biosynthesis is reorganized in the *tab2* mutant under those conditions, with aromatic and branched chain amino acid biosynthesis being shut down in favor of maintenance of pools of amino acids that are closely connected to the citrate cycle and that could quickly respond to enable assimilation of N, should a new source of that critical nutrient become available in the environment surrounding the cell. That is similar to what occurs in WT cells [41]. However, in the *tab2* mutant succinate connects the cytosolic and peroxisomal glyoxylate cycles to the citrate cycle, supporting this potential assimilation priming. In the WT cells, TAG is produced at this stage of N starvation, to serve both as a storage molecule and potentially as a mechanism to protect the cell from oxidative damage associated with the N starvation state, leading eventually to entrance into the quiescent state outlined above. The *tab2* cells, on the other hand, suffer from the inability both to produce TAG and to protect themselves from oxidative damage, due to reduced NADPH availability.

The increased acetate utilization by the *tab2* cells compared to the WT does not increase TAG accumulation, supporting the idea that TAG biosynthesis in Chlamydomonas may play more of a role in regulating the energy balance in the cell than in serving as a carbon sink. Likewise, the GND and GLD enzymes involved in NADPH production by the oxidative pentose phosphate pathway are highly up-regulated in the mutant (Additional file 15: Figure S14C) indicating that NADPH produced through the oxidation of the G6P may not contribute to the TAG accumulation process in Chlamydomonas. These findings support the conclusion that TAG synthesis in Chlamydomonas either depends on active photosynthesis or plays a role that is greater than just generic bulk carbon storage. It is thus likely that the knockdown of TAB2 causes a severe photo-oxidative stress in the mutant followed by the production of ROS. Although we did not measure augmentation in vivo, we found that the enzymes involved against ROS are highly up-regulated in the mutant versus WT, which is not surprising. This result suggests that the TAG biosynthesis could involve protecting the photosynthetic machinery from the photodamage through the regeneration of NADP+ which can accept electrons and thus reduce the production of ROS.

Thus, efforts to increase lipid accumulation in algae such as Chlamydomonas need to consider carefully how the energy balance of the cell is involved in or affected by such efforts and that numerous layers of metabolic and genetic regulatory control are likely interfered with such efforts to control oil biosynthesis.

## Conclusions

In this study, we conducted a detailed physiological and omics-based analysis of an insertional mutant of Chlamydomonas with reduced TAB2 function to determine what role this protein may play in regulating the cellular response to N deprivation. TAB2 is a photosystem I (PSI) translation initiation factor, whose transcript and protein levels increase significantly after only 30 min of N deprivation. The *tab2* mutant displayed reduced TAG content and altered fatty acid profiles, along with increased starch and acetate assimilation during the first 6 h of N deprivation, followed by a metabolites shift towards amino acid synthesis, revealing the central role of functional PSI in controlling lipid/starch partitioning. A time course analysis of the ATP/NADPH ratio in the mutant vs. wild type suggested that TAG biosynthesis may be associated with maintaining the redox state of the cell during N deprivation. The loss of ability to accumulate TAG in the *tab2* mutant co-occurred with an up-regulation of photo-protective mechanism and autophagy, suggesting that the synthesis of TAG in the wild type occurs not only as a temporal energy sink, but also as a protective mechanism. Such knowledge will enable synthetic biology approaches to alter the response to the N depletion stress, leading to rewiring of the regulatory networks so that lipid accumulation could be turned on in the absence of N deprivation, allowing for the development of algal production strains with highly enhanced lipid accumulation profiles.

## Additional files

**Additional file 1: Table S1.** The list of primers used for the quantitative real-time PCR results for the WT and *tab2*.

**Additional file 2: Figure S1.** Key for visualization of protein expression levels via heat maps of chlorophyll and carotenoids biosynthesis. In heat maps protein expression levels of all conditions (WT at time 0, WT after 48 h of N deprivation, *tab2* at time 0 and *tab2* after 48 h of N deprivation) are compared. The shown mean ratios are log2.

**Additional file 3: Figure S2.** Correlation diagram of TAG content/ Starch and Cell size area during N deprivation in the WT (A) and in *tab2* (B), Correlation of starch content with cell area size is represented with solid triangles and dash line. Correlation of TAG content with cell area size is represented with solid circles.

**Additional file 4: Figure S3.** Key for visualization of protein expression levels via heat maps of photosystem complexes. In the heat maps, protein expression levels of all conditions (WT at time 0, WT after 48 h of N deprivation, *tab2* at time 0 and *tab2* after 48 h of N deprivation) are compared. The shown mean ratios are log2.

**Additional file 5: Figure S4.** Key for visualization of protein expression levels via heat maps of mitochondrial electron transport mechanisms. In the heat maps, protein expression levels of all conditions (WT at time 0, WT after 48 h of N deprivation, *tab2* at time 0 and *tab2* after 48 h of N deprivation) are compared. The shown mean ratios are log2.

**Additional file 6: Figure S5.** The 77K Steady State Fluorescence Emission Spectra of C. reinhardtii WT Cells grown in TAP media during N deprivation. The amplitude of the PSII-associated signal (around 685 nm) and the PSI-associated signal (around 715 nm).

**Additional file 7: Figure S6.** Detailed Lipid analysis of the wild-type and *tab2* in N+ conditions. (A) Changes in TAG content in the wild-type and *tab2*. (B) Mol (%) of esterified fatty acids in TAG isolated from N+ condition cells in the Wild-type (C) and in *tab2* (D). Time points presented are 0, 2, 4, 6 and 8 days. Values are representative of triplicate biological samples. Error bars indicate *SE* means.

**Additional file 8: Figure S7.** Visualization of protein expression levels for fatty acid biosynthesis. Heat maps compare protein expression levels relative to wild type time 0 (WT 0h), including WT after 48 h of N deprivation, *tab2* at time 0 and *tab2* after 48 h of N deprivation. The ratios are displayed in log2 scale.

**Additional file 9: Figure S8.** Quantitative real-time PCR Results for WT and *tab2*. The transcript level was represented by white bars for the WT and black bars for the mutant. Time points presented are 0.5, 2, 6, 24, 72 h.

**Additional file 10: Figure S9.** Key for visualization of protein expression levels via heat maps of (A) gluconeogenesis and starch biosynthesis and (B) glycolysis and starch catabolism. In the heat maps, protein expression levels of all conditions (WT at time 0, WT after 48 h of N deprivation, *tab2* at time 0 and *tab2* after 48 h of N deprivation) are compared. The shown mean ratios are log2.

**Additional file 11: Figure S10.** Key for visualization of protein expression levels via heat maps of (A) acetate uptake and (B) central metabolism. In the heat maps, protein expression levels of all conditions (WT at time 0, WT after 48 h of N deprivation, *tab2* at time 0 and *tab2* after 48 h of N deprivation) are compared. The shown mean ratios are log2.

**Additional file 12: Figure S11.** Metabolic profiles of the WT and the *tab2*. Time points presented are 0, 0.5, 2, 6, 24, 48, 72, 96, 120, 144 h. Data are normalized to the means' standard deviations. Circles correspond to the WT profile. Squares corresponded to the *tab2* profile.

**Additional file 13: Figure S12.** Key for visualization of protein expression levels via heat maps of amino acids and polyamines. In the heat maps, protein expression levels of all conditions (WT at time 0, WT after 48 h of N deprivation, *tab2* at time 0 and *tab2* after 48 h of N deprivation) are compared. The shown mean ratios are log2.

**Additional file 14: Figure S13.** Time-course amino acids profile during N deprivation in the WT and *tab2*. Time points presented are 0, 0.5, 2, 6, 24, 48, 72, 96, 120, 144 h. Data are normalized to the means standard deviation. Squares corresponded to the WT profile. Circles corresponded to the *tab2* profile.

**Additional file 15: Figure S14.** Key for visualization of protein expression levels via heat maps of (A) ROS protection, (B) proteolysis and (C) OPPP. In the heat maps, protein expression levels of all conditions (WT at time 0, WT after 48 h of N deprivation, *tab2* at time 0 and *tab2* after 48 h of N deprivation) are compared. The shown mean ratios are log2.
