# Supplementary material for: Functional photosystem I maintains proper energy balance during nitrogen depletion in Chlamydomonas reinhardtii, promoting triacylglycerol accumulation
Source: Biotechnol Biofuels. 2017 Apr 13;10:89. doi: 10.1186/s13068-017-0774-4 (PMC5390395; doi:10.1186/s13068-017-0774-4)
Supplement: Supplementary file 1 — Additional file 1: Table S1. The list of primers used for the quantitative real-time PCR results for the WT and tab2. [file 13068_2017_774_MOESM1_ESM.docx]

| Primer name | Forward sequence | Reverse sequence |
| --- | --- | --- |
| ACX1 | G C A T C C T C A A C G T C T A C T C C | T T G A C C A C A A A C T C T T G G A A |
| BCX1 | C G T C A T G G A T T T C A C C T A C A | G A G G T G C A C A C A A T G A T G A C |
| BCC1 | C T A A G A C C G A G G A G G T T G A G | A C A A T G T C G G T A T C A T G C A C |
| BCC2 | A G G T T G A G G A G G A G T T C G A C | A T C T T C A G G T G C A T C T C A G C |
| ACP1 | C C G A C C T T C A C C A A C A T A A T | T T G G A T G C A T C A A T C T T C T C |
| ACP2 | C A C T G A G C T G G A G A A G G T T G | C T C C T C C A G G G C A A T C T C |
| DGAT1 | C A A G A A G C T C A A C A G A G A C G | C C A C G A C G T A G T C A T G G T A A |
| DGTT1 | G C C T T C C T G C T G T A C C T C T A | T A A G A G G C C A T A A C C T T C C A |
| DGTT2 | T A C G T G T T T G A T G A G G T G C T | A C A T G A T C T G G C A T T C T G T G |
| DGTT3 | G A T G T A C A T G G G C A A C A A G A | A C C G A A G T A G T A C A C G C A C A |
| DGTT4 | G T T C G T G C A G T T C A G T G T G | G G C A G A A T C C G A A C A A G T A G |
| DGTT5 | C A C T G G G T T T G C T A G T A C C G | T A G T G G T G A T G G T G G C A G T A |
| PDAT1 | C G A C A G A G T C A A G G A C T C A A | G C A T A C C A T T G T T G T C T T G G |
| TAB2 | C T G G G A G A T C G A C T T C T G T T | G A T C T T G C T G T T C G G A A A G T |
| PGI1 | G C T C A G G T C G C T A A A A T G A T | A G G T G C G T C T T C T C A A T C T C |
| GPM1 | G C T A C G A T T A C G A G G A G T G C | G G G T C C G T G T A C T C A A A G T C |
| GPM2 | A G G C A T G T A C A A G G A C A A G G | A G T C A A A G A A G C C G T A C C T G |
| AGPase1 | G A C G G T G C T G G G T A T T A T T C | A G T T G C T A A C G G G A A T A T C G |
| AGPase2 | G A C T T C G G T G G T G A G A T C A T | C T C C T C G A A G A A G G A C T T G A |
| SSS1 | T G C T C A C C T A C A A C A A C C A C | C A A T G A T C T G C T C G T A C T G C |
| SSS2 | C C C G G T A T G A G A A C T A C G A C | G T C C A C A A A C A C G T A G T C C A |
| SSS3 | G G A G A C G T A C G A G C T C A A C T | A C C C A G G T G T C G T A G T C A A T |
| SSS4 | T G G A G G C C T A C C T G T A C T T C | T A C A C G T C C C A G T A C A G C A T |
| SSS5 | A C G A C G T T A A G G A G C T G A A G | A G T T G G G C T C G A T G A A G T A G |
| SBE1 | G C T G T C C A A C C T G A G G T A C T | T G C C C A G A T A C T C G T T G T A G |
| SBE2 | G G C T A T T A T G G A G C T G G A G A | A G C G G T C C T T G T A C T C G T A G |
| SBE3 | T A C G G C A A C T G G G A G A C T A T | G T G G T A C A T C A T C G A G G T C A |
| PGD 1 | G G A T G T C A G C G G A T T T C T T | C A C C A G G T G C A T G T A G T A G G |
| MAS1 | G A C C A G C T G A A G A G C A A G A T | G T G G T G A G G A A G T C C G A G T A |
| ICL1 | G A G T A C G A C A A G C C C T T C A T | G G T C T C A A A C C A C A C C A G A T |
| PCK 1 | T C T G G A G A A C G T G G A C T A C A | G G C G T T G T T C A T A A A C T C A A |
| PPDK 1 | G A G G T C T G C G A G G A G T T C T A | G T G T T G A C G T C T C C G A A C T T |
| FBP1 | G C A A C T A C A T C G T G G T G T T C | C A C T G C T C C A T C A T C T T C T G |
| FBP2 | G G C T A C G A C C T G A A C A A G A T | A T G A C G A T G C T G T T G G T G |
| FSD1 | C C A T C C T G A C T G T C G A T G T G | C C C A G T T G A T C A G C T T C T C C |
| GPD1 | A G C C G C A A A G A A A A T T G T A G | T G A T G T C C T T G C A C A G G T A G |
| GPD2 | G C G G T A C G C T C A A G A A T A T C | C A G A A G T C A C G C A T C T C A A G |
| GPD3 | G T A C G C T C A A G A A C A T C G T G | C A G A A G T C A C G C A T C T C A A G |
| GPD4 | T A C G A C T A C A C G G A C C T C C T | C A C T T C G T C C A C A A A C T C A T |
| GPD5 | C C C A C G C A G T A C A T C A T A C A | G T C T C C A G G C T G T C A A G A C T |
| G6PD | G G A G G A G T T C C A C A T C T T T G | G T C T T C T C C T G G C A G T T C T C |
| 6PGDH | T T C C C A T T T C G G T C T A C A A C | A A A G T C C T T G A C T T G C T C G T |
| MME5 | C A C C T T C A A C G A C G A C A T C | A A G A A C A G G A A G G T C T G C T G |
| MDH | A G T T C T A C A C C T C G G T C A G C | C A C T T C A T G T C A C G G A T C A C |
| APX1 | G C G T G T T A G T G C A A A G G T C A | G G G A A C T C A G C G A T G T T C T T |
| APX2 | A G C C C T G G A A C A A C A C A A A G | A G A A G T C G C G G A A G A A C A G A |
| PKY1 | A G T A G C A G C G G C A T T A G C T C | A C C G T T G T G A T T G G G G A T T A |
| PGM2 | C C T A C C T G C T T C A T G G C A A C | C T C A G C A T T C C A G T C G G T C T |
| PGM3 | C T G C A G G T A C C A G T G C A G A C | C A T C A C G C T C C C A T A T T G T G |
| PGM4 | T A C A G T A C G G C A A C G T G C T C | G C A A A C T C A C A C A C C G A C A G |
| PGM6 | C G C T T C A A G C A T T T C C T G T A | C G C A G T G G T C C A T G T A G T T G |
| MSD1 | C A C C A C C A G A C C T A C G T G A A | T G A C A G T G G C G A C A T C C T T A |
| MSD2 | A C T A T G A C C C G C G C T A T G T C | G C C T T G T T C A G G T T C G C T A C |
| MSD3 | T C G G A G A T T G T C A G C C A G A T | T G G A G G A G T T G A A G G T G G T C |
| GPX3 | A A G T C C A A C T A C G G C G T G A C | A G A C C A C C C T T C T G G G T C T T |
| GDH1 | C A A G A T T G T G G G C G T A T C C T | G A A T G A G T C G T C G T T G A G C A |
| CAT2 | A T C A A G T G G G A C G C T G A G A T | C G T C G T C G T T A C A C A G G T T G |

**Additional file 1: Table S1**
