# Supplementary figures and images for: Functional photosystem I maintains proper energy balance during nitrogen depletion in Chlamydomonas reinhardtii, promoting triacylglycerol accumulation
Source: Biotechnol Biofuels. 2017 Apr 13;10:89. doi: 10.1186/s13068-017-0774-4 (PMC5390395; doi:10.1186/s13068-017-0774-4)

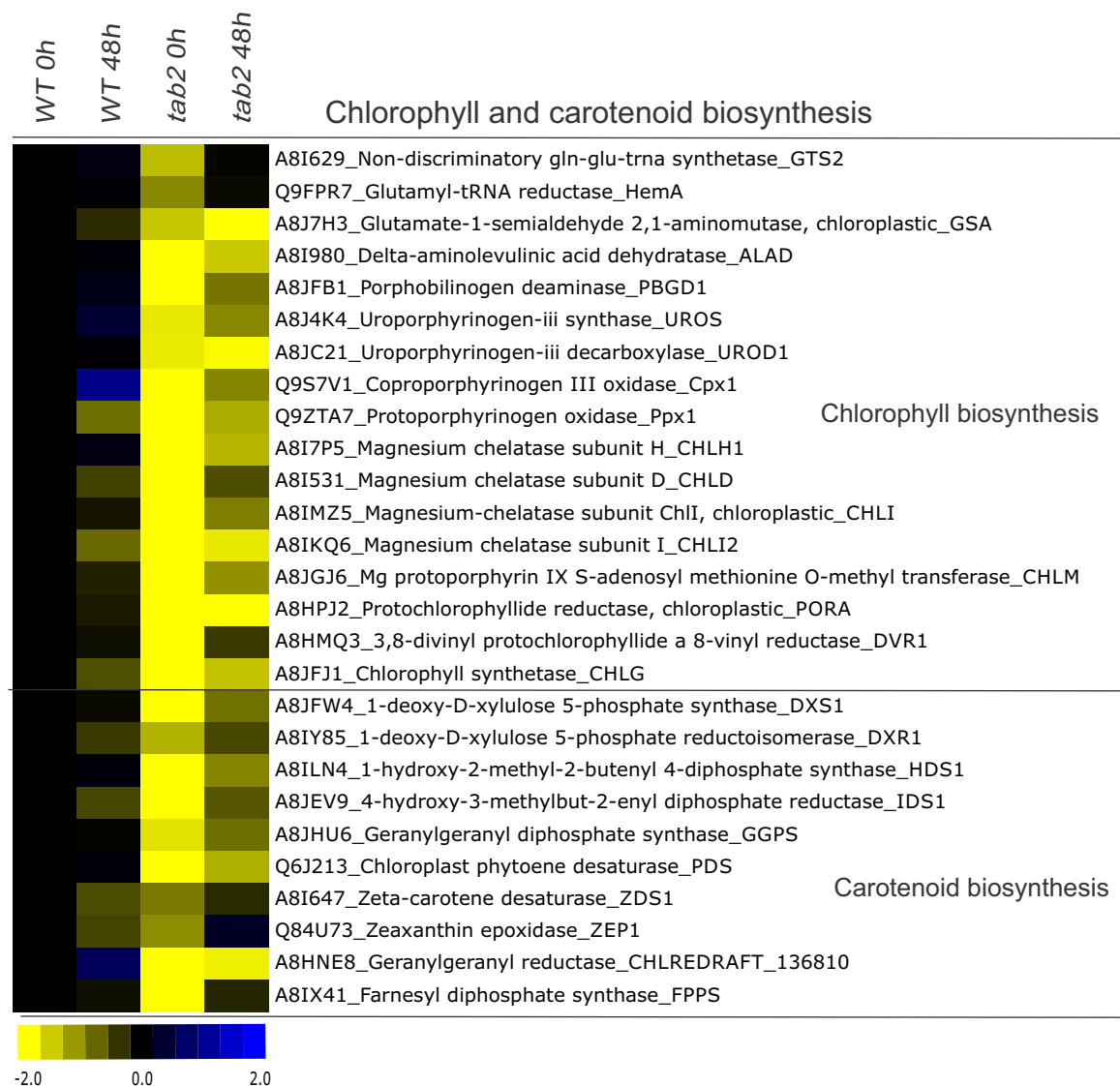

Additional file 2: Figure S1.

Supplement: Supplementary file 2 — Additional file 2: Figure S1. Key for visualization of protein expression levels via heat maps of chlorophyll and carotenoids biosynthesis. In heat maps protein expression levels of all conditions (WT at time 0, WT after 48 h of N deprivation, tab2 at time 0 and tab2 after 48 h of N deprivation) are compared. The shown mean ratios are log2. [file 13068_2017_774_MOESM2_ESM.pdf]

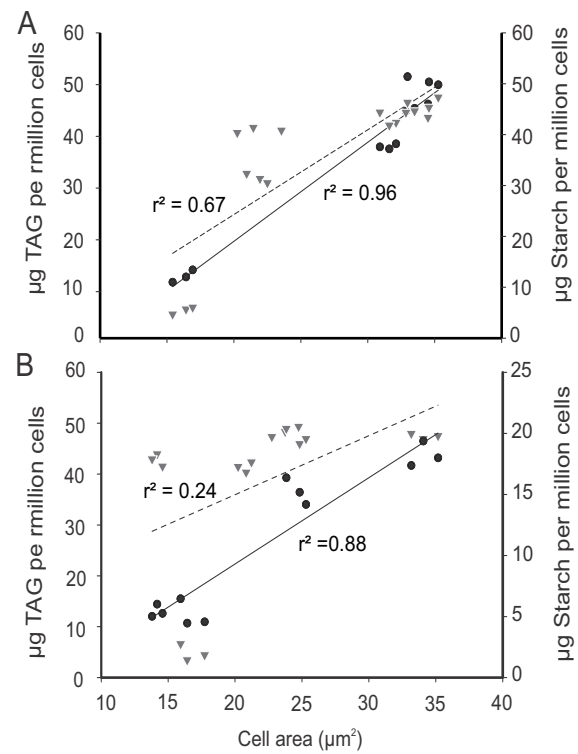

Additional file 3: Figure S2.

Supplement: Supplementary file 3 — Additional file 3: Figure S2. Correlation diagram of TAG content/ Starch and Cell size area during N deprivation in the WT (A) and in tab2 (B), Correlation of starch content with cell area size is represented with solid triangles and dash line. Correlation of TAG content with cell area size is represented with solid circles. [file 13068_2017_774_MOESM3_ESM.pdf]

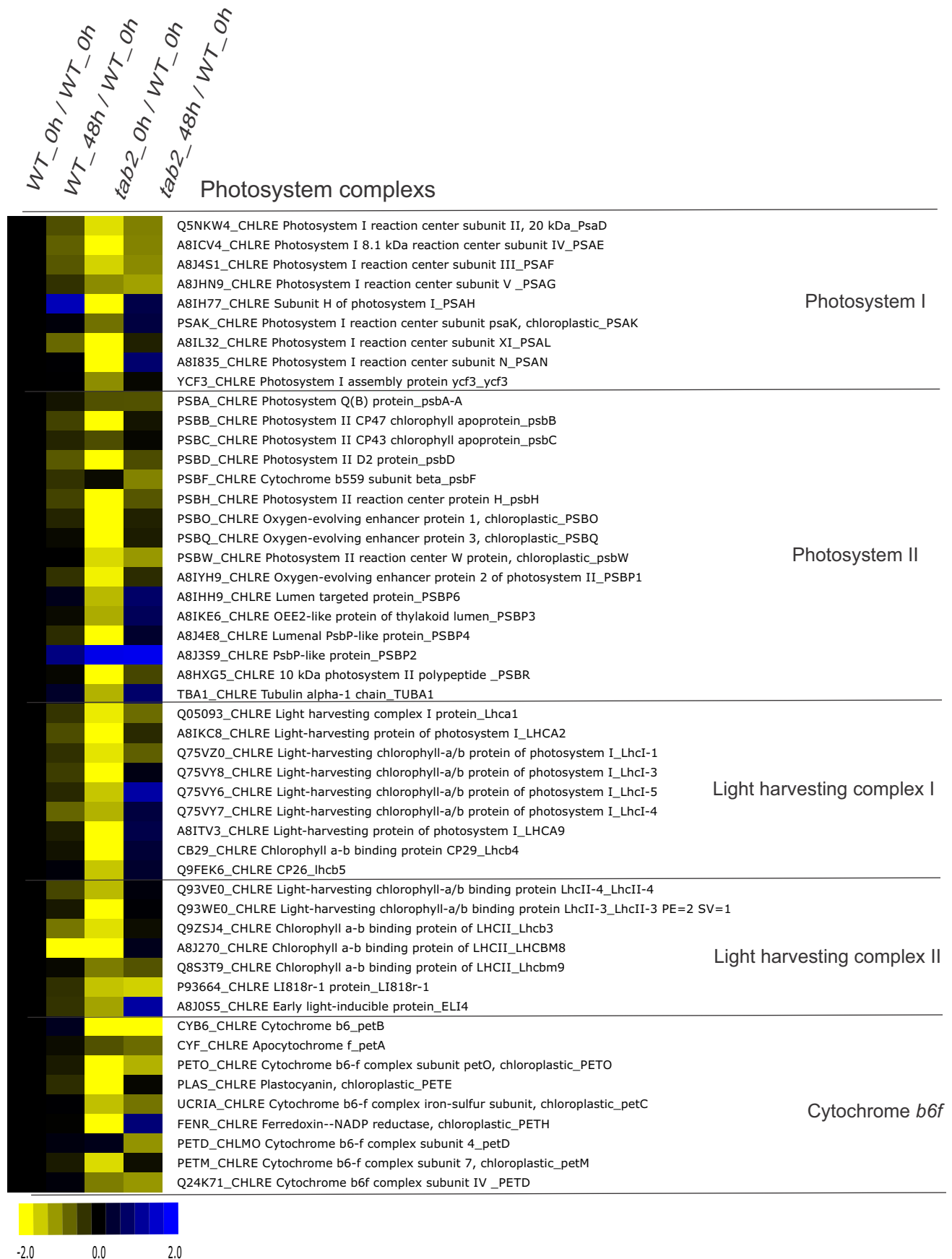

Additional file 4: Figure S3.

Supplement: Supplementary file 4 — Additional file 4: Figure S3. Key for visualization of protein expression levels via heat maps of photosystem complexes. In the heat maps, protein expression levels of all conditions (WT at time 0, WT after 48 h of N deprivation, tab2 at time 0 and tab2 after 48 h of N deprivation) are compared. The shown mean ratios are log2. [file 13068_2017_774_MOESM4_ESM.pdf]

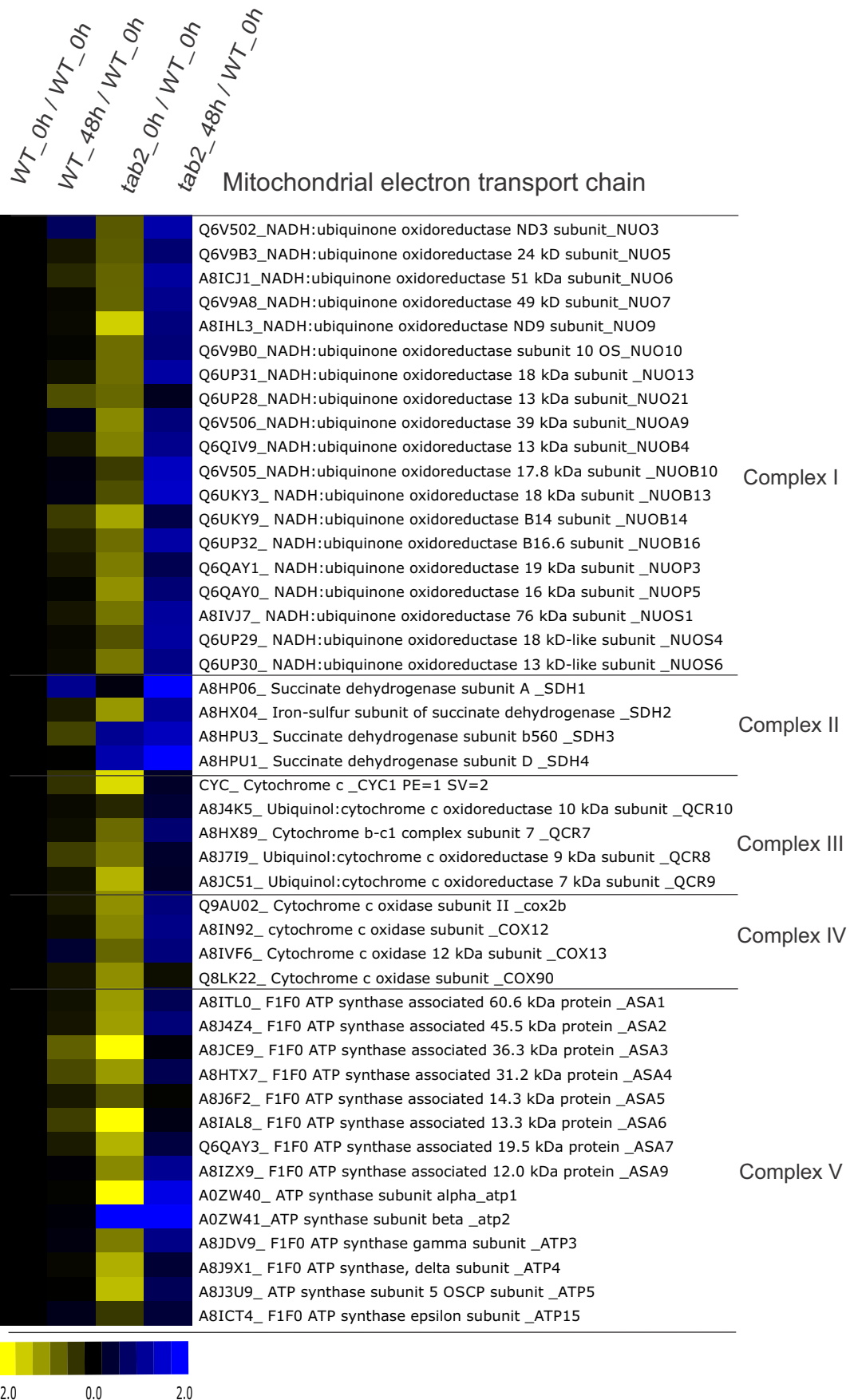

Additional file 5: Figure S4.

Supplement: Supplementary file 5 — Additional file 5: Figure S4. Key for visualization of protein expression levels via heat maps of mitochondrial electron transport mechanisms. In the heat maps, protein expression levels of all conditions (WT at time 0, WT after 48 h of N deprivation, tab2 at time 0 and tab2 after 48 h of N deprivation) are compared. The shown mean ratios are log2. [file 13068_2017_774_MOESM5_ESM.pdf]

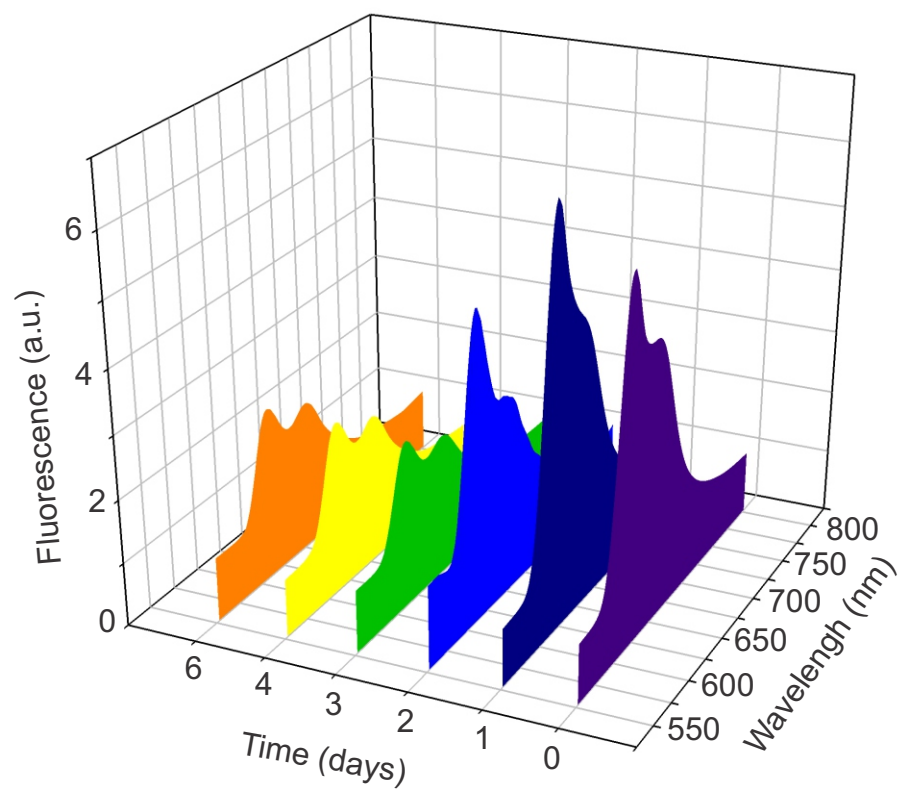

Additional file 6: Figure S5.

Supplement: Supplementary file 6 — Additional file 6: Figure S5. The 77K Steady State Fluorescence Emission Spectra of C. reinhardtii WT Cells grown in TAP media during N deprivation. The amplitude of the PSII-associated signal (around 685 nm) and the PSI-associated signal (around 715 nm). [file 13068_2017_774_MOESM6_ESM.pdf]

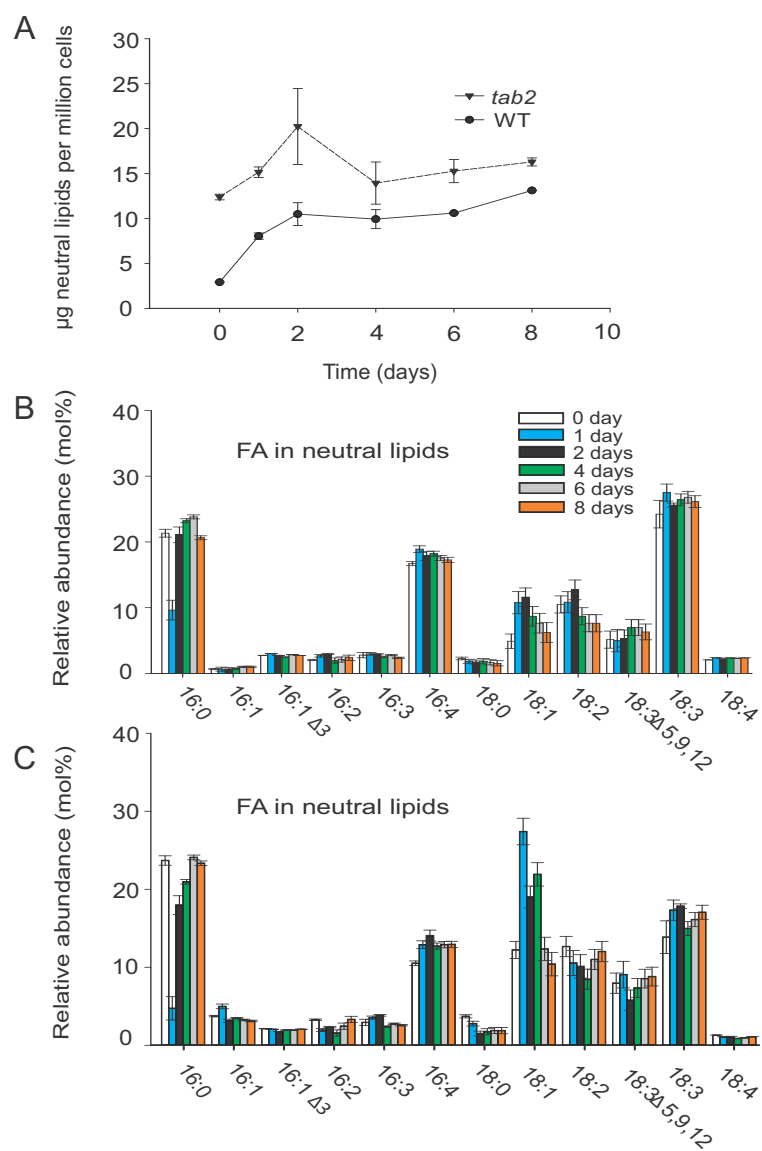

Additional file 7: Figure S6.

Supplement: Supplementary file 7 — Additional file 7: Figure S6. Detailed Lipid analysis of the wild-type and tab2 in N+ conditions. (A) Changes in TAG content in the wild-type and tab2. (B) Mol (%) of esterified fatty acids in TAG isolated from N+ condition cells in the Wild-type (C) and in tab2 (D). Time points presented are 0, 2, 4, 6 and 8 days. Values are representative of triplicate biological samples. Error bars indicate SE means. [file 13068_2017_774_MOESM7_ESM.pdf]

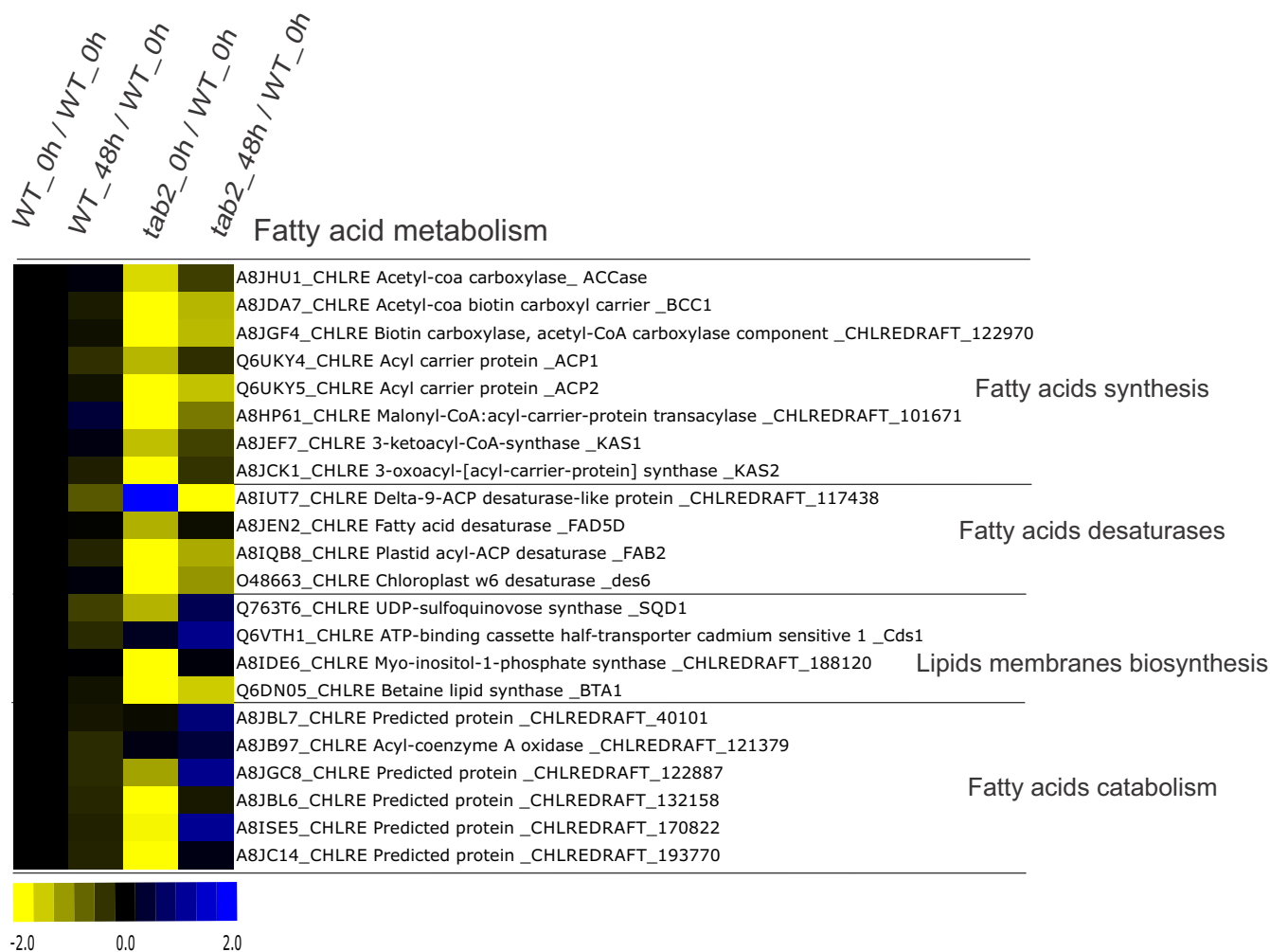

Additional file 8: Figure S7.

Supplement: Supplementary file 8 — Additional file 8: Figure S7. Visualization of protein expression levels for fatty acid biosynthesis. Heat maps compare protein expression levels relative to wild type time 0 (WT 0h), including WT after 48 h of N deprivation, tab2 at time 0 and tab2 after 48 h of N deprivation. The ratios are displayed in log2 scale. [file 13068_2017_774_MOESM8_ESM.pdf]

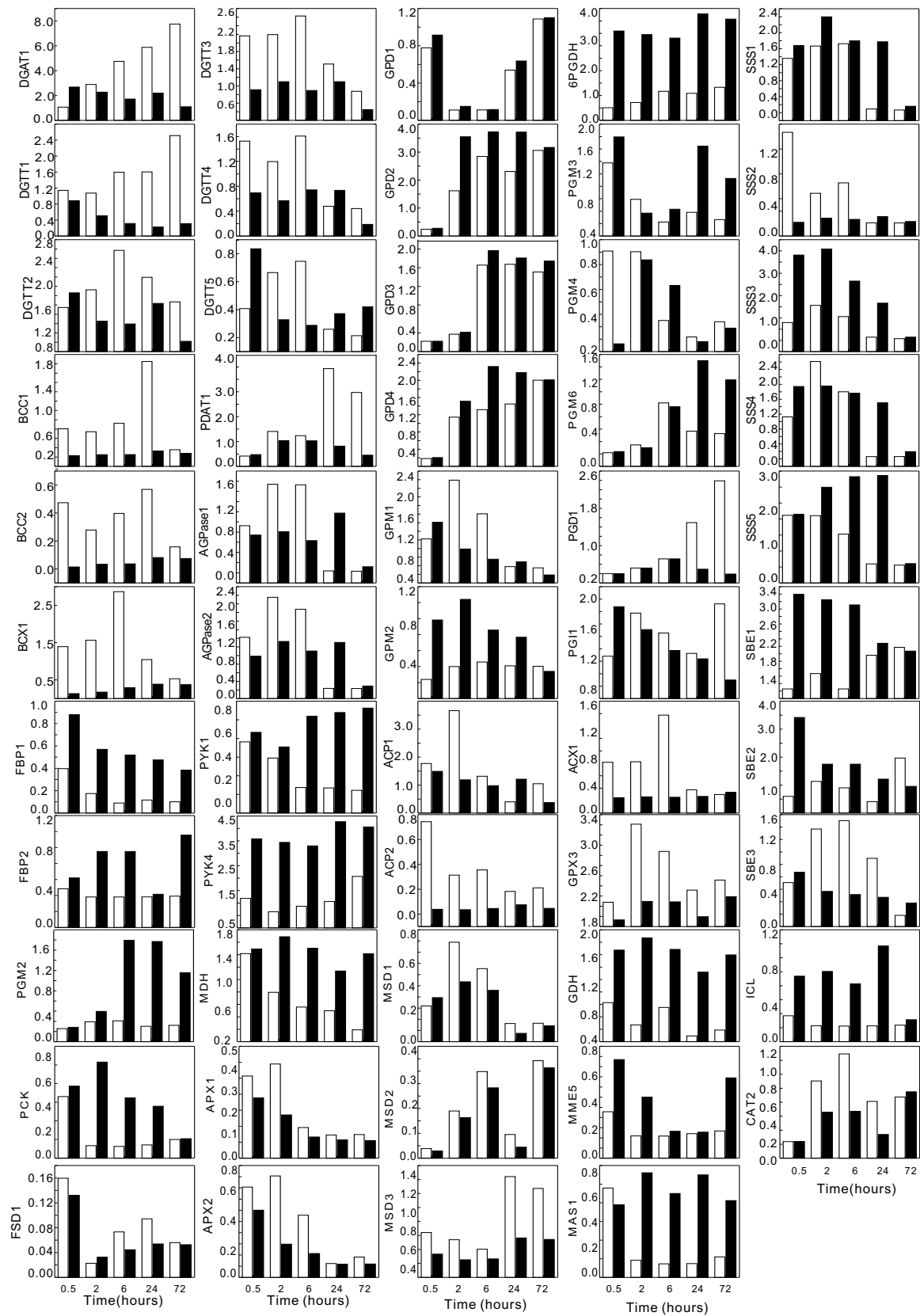

Additional file 9: Figure S8.

Supplement: Supplementary file 9 — Additional file 9: Figure S8. Quantitative real-time PCR Results for WT and tab2. The transcript level was represented by white bars for the WT and black bars for the mutant. Time points presented are 0.5, 2, 6, 24, 72 h. [file 13068_2017_774_MOESM9_ESM.pdf]

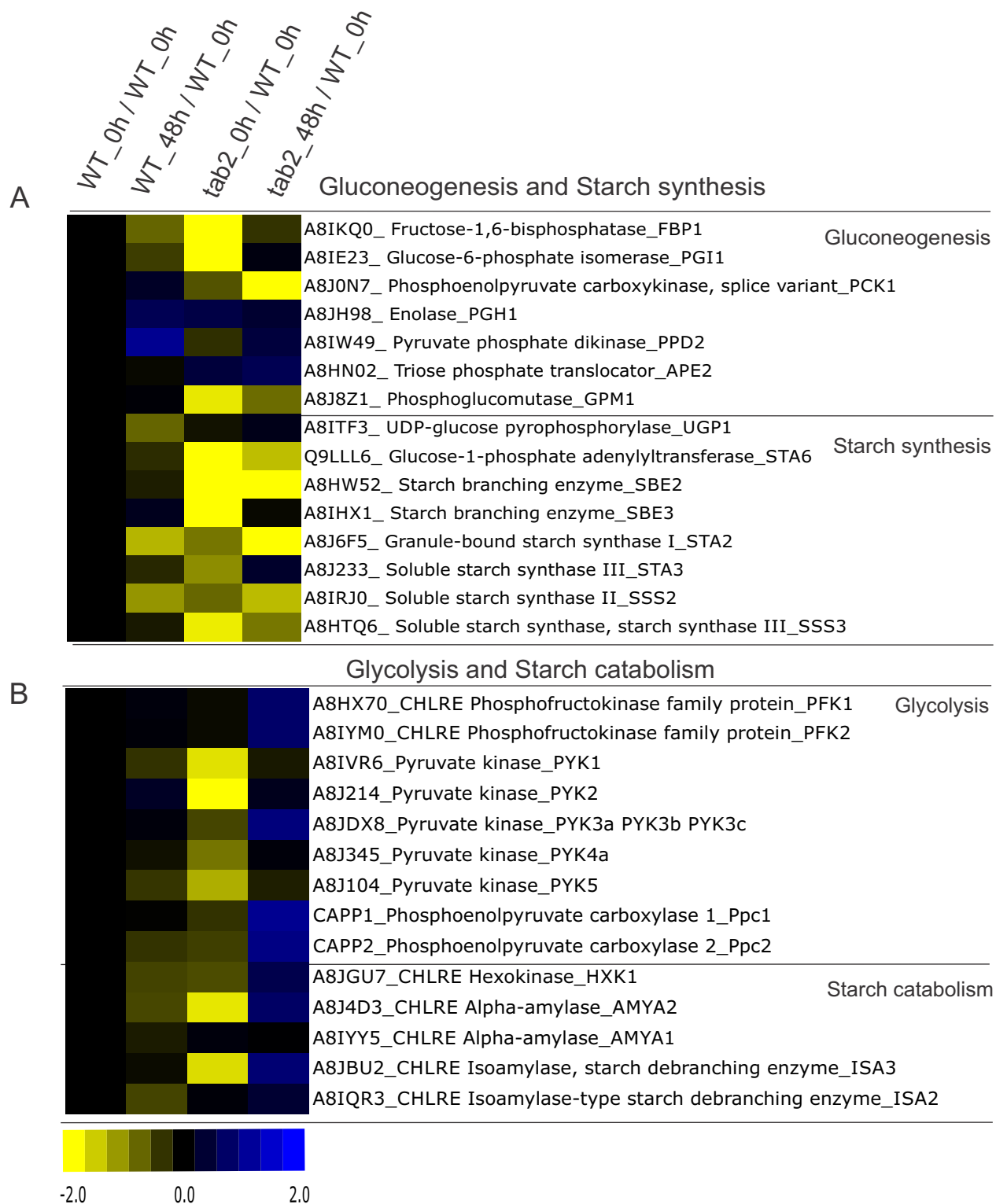

Additional file 10: Figure S9.

Supplement: Supplementary file 10 — Additional file 10: Figure S9. Key for visualization of protein expression levels via heat maps of (A) gluconeogenesis and starch biosynthesis and (B) glycolysis and starch catabolism. In the heat maps, protein expression levels of all conditions (WT at time 0, WT after 48 h of N deprivation, tab2 at time 0 and tab2 after 48 h of N deprivation) are compared. The shown mean ratios are log2. [file 13068_2017_774_MOESM10_ESM.pdf]

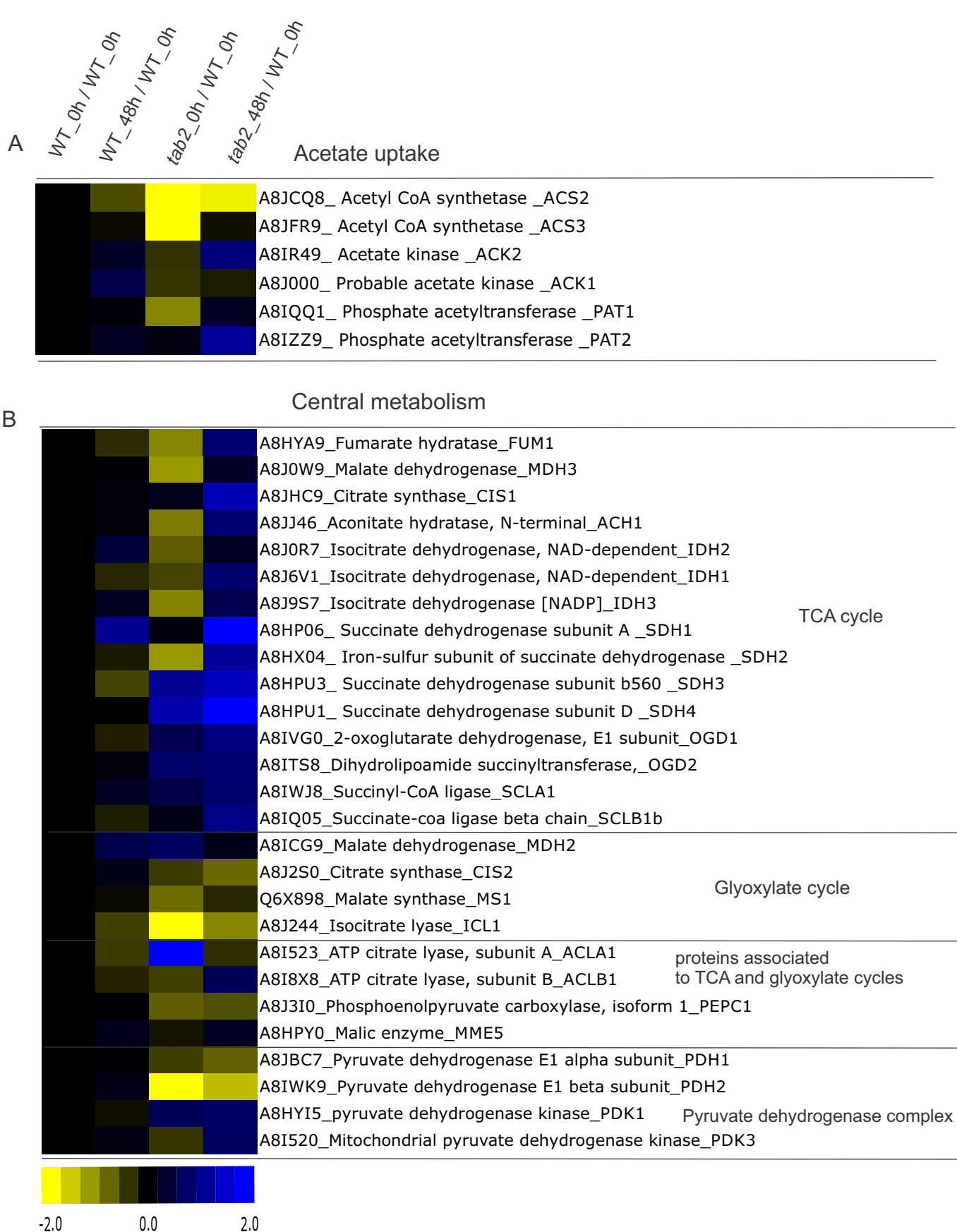

Additional file 11: Figure S10.

Supplement: Supplementary file 11 — Additional file 11: Figure S10. Key for visualization of protein expression levels via heat maps of (A) acetate uptake and (B) central metabolism. In the heat maps, protein expression levels of all conditions (WT at time 0, WT after 48 h of N deprivation, tab2 at time 0 and tab2 after 48 h of N deprivation) are compared. The shown mean ratios are log2. [file 13068_2017_774_MOESM11_ESM.pdf]

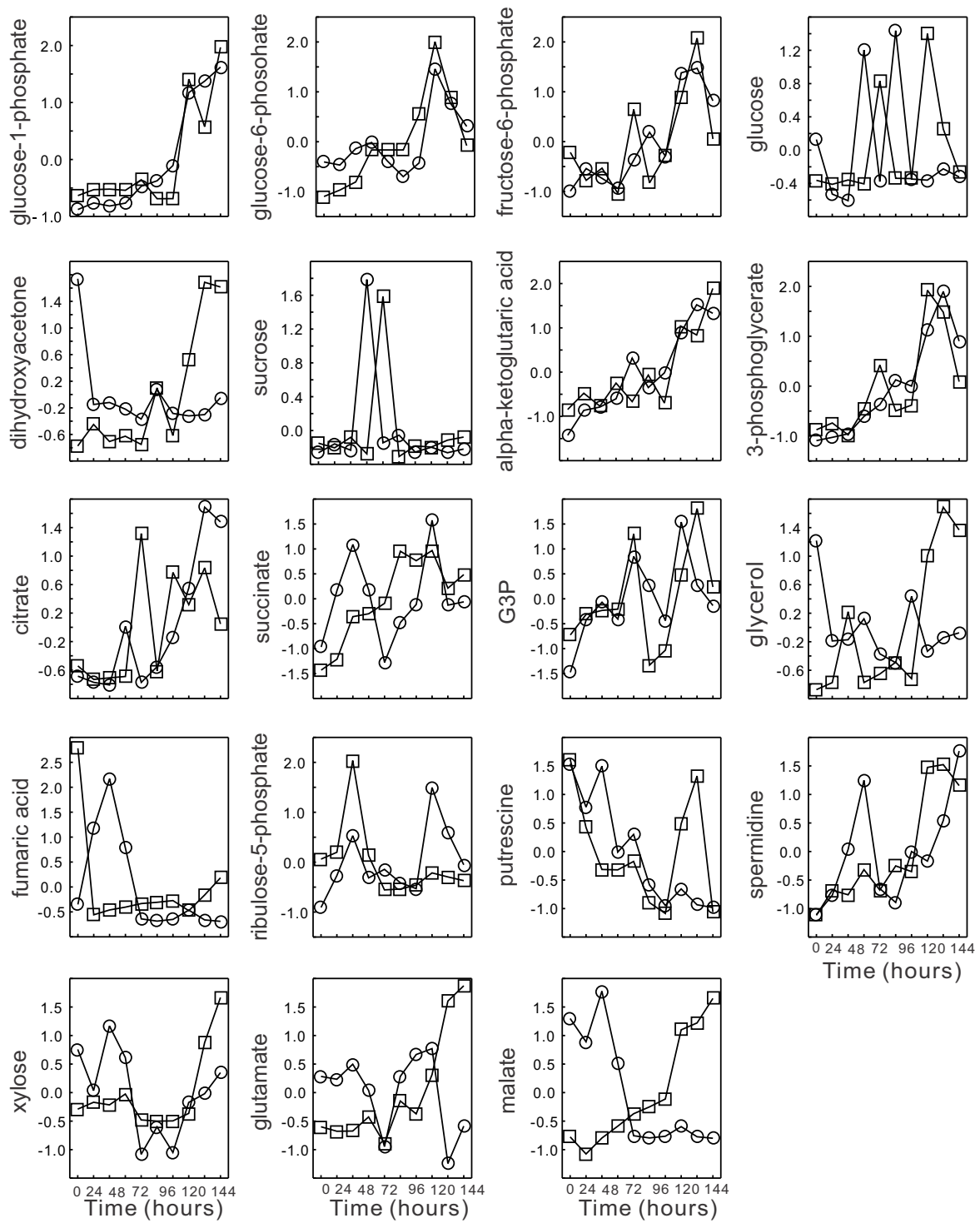

Additional file 12: Figure S11.

Supplement: Supplementary file 12 — Additional file 12: Figure S11. Metabolic profiles of the WT and the tab2. Time points presented are 0, 0.5, 2, 6, 24, 48, 72, 96, 120, 144 h. Data are normalized to the means' standard deviations. Circles correspond to the WT profile. Squares corresponded to the tab2 profile. [file 13068_2017_774_MOESM12_ESM.pdf]

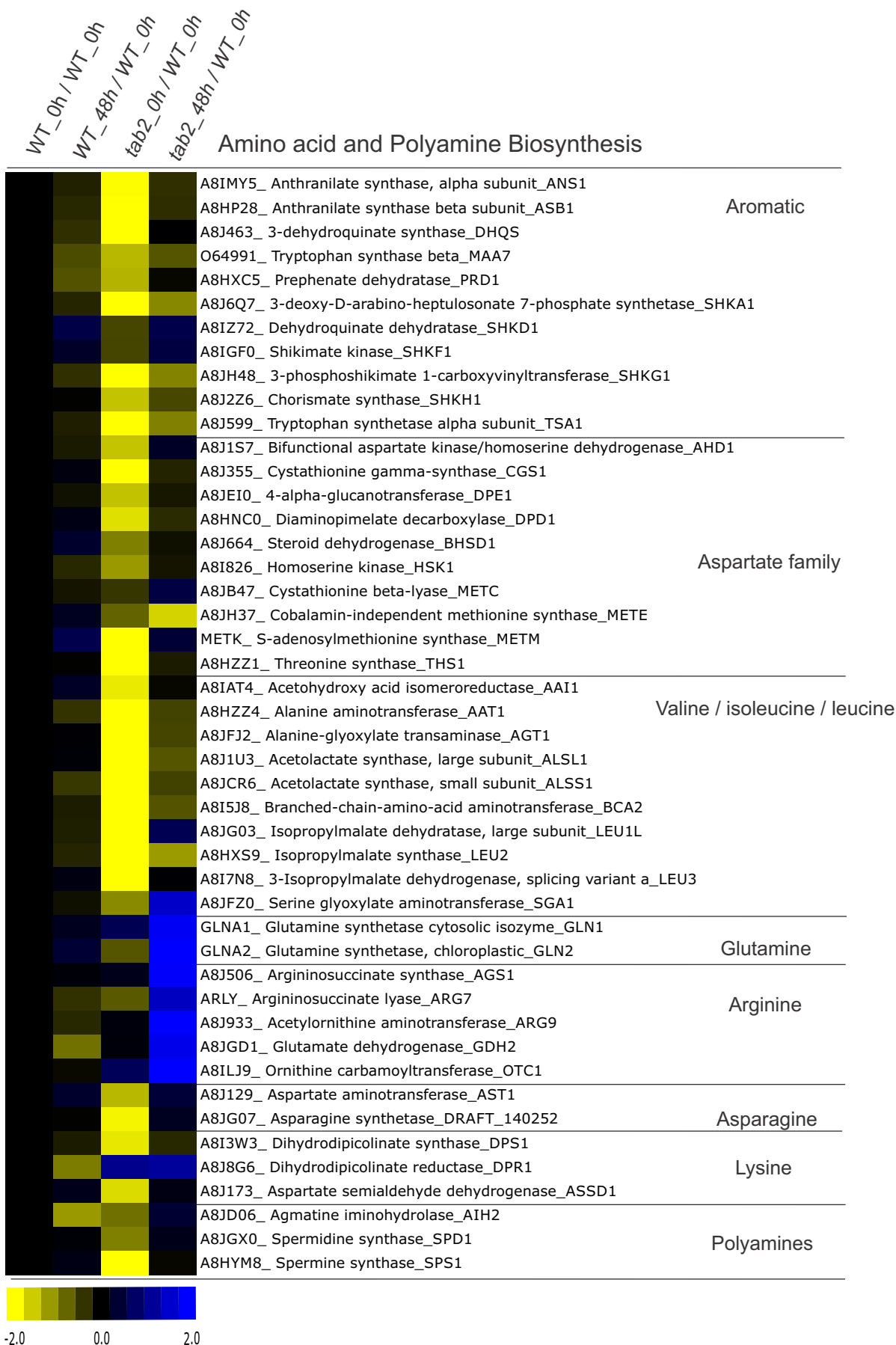

Additional file 13: Figure S12.

Supplement: Supplementary file 13 — Additional file 13: Figure S12. Key for visualization of protein expression levels via heat maps of amino acids and polyamines. In the heat maps, protein expression levels of all conditions (WT at time 0, WT after 48 h of N deprivation, tab2 at time 0 and tab2 after 48 h of N deprivation) are compared. The shown mean ratios are log2. [file 13068_2017_774_MOESM13_ESM.pdf]

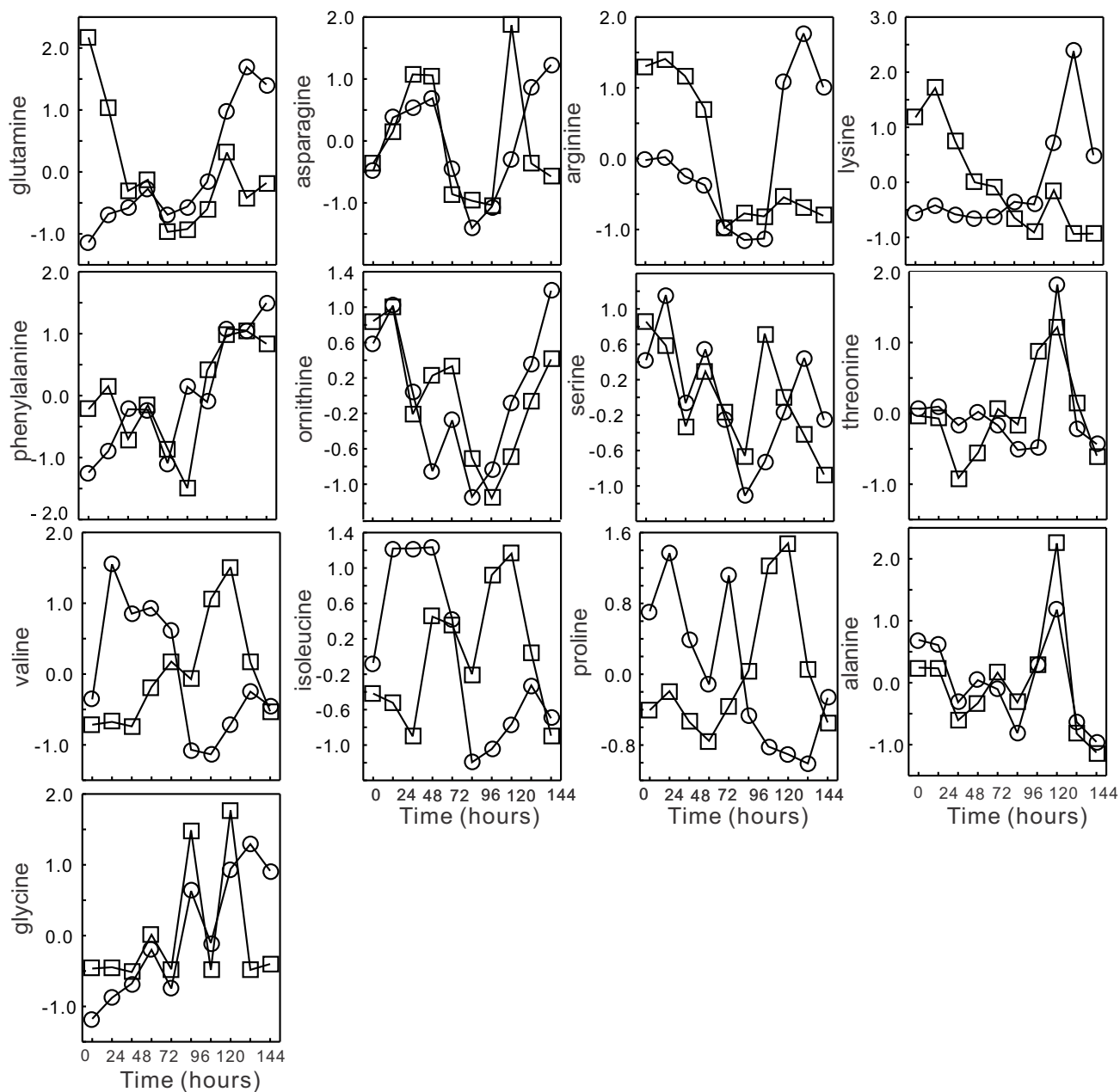

Additional file 14: Figure S13.

Supplement: Supplementary file 14 — Additional file 14: Figure S13. Time-course amino acids profile during N deprivation in the WT and tab2. Time points presented are 0, 0.5, 2, 6, 24, 48, 72, 96, 120, 144 h. Data are normalized to the means standard deviation. Squares corresponded to the WT profile. Circles corresponded to the tab2 profile. [file 13068_2017_774_MOESM14_ESM.pdf]

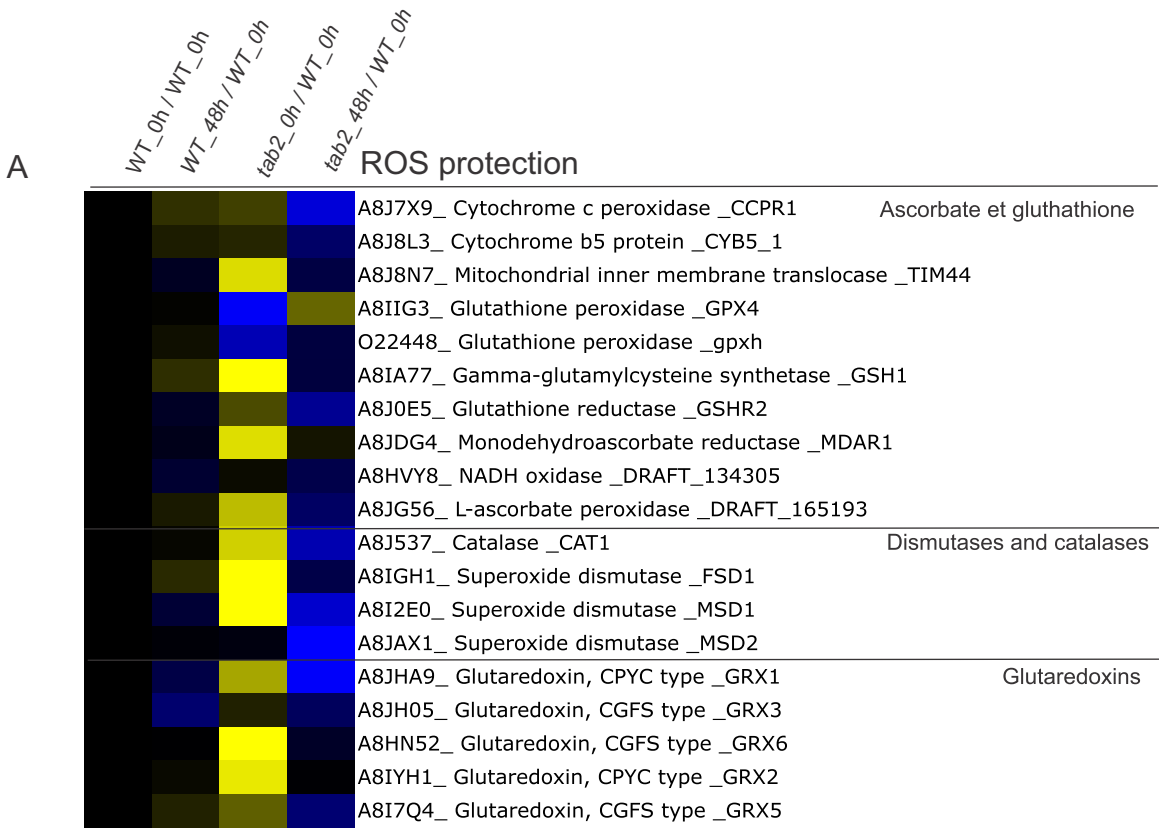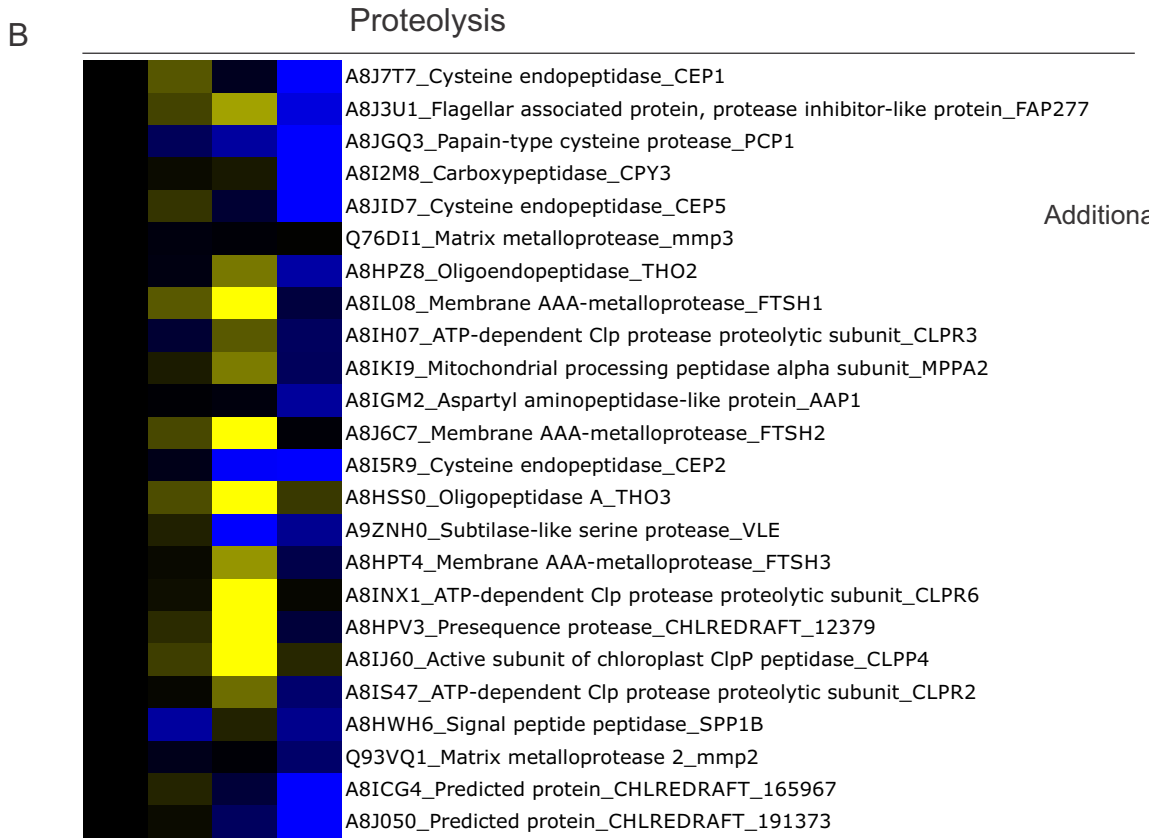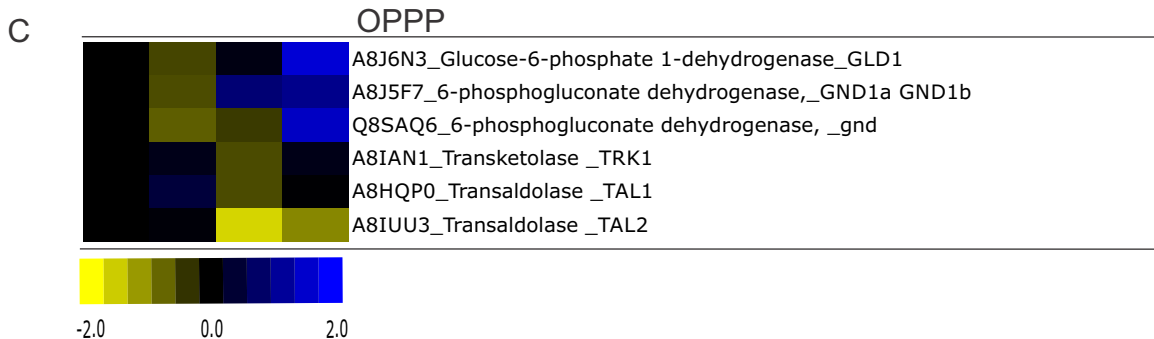

Supplement: Supplementary file 15 — Additional file 15: Figure S14. Key for visualization of protein expression levels via heat maps of (A) ROS protection, (B) proteolysis and (C) OPPP. In the heat maps, protein expression levels of all conditions (WT at time 0, WT after 48 h of N deprivation, tab2 at time 0 and tab2 after 48 h of N deprivation) are compared. The shown mean ratios are log2. [file 13068_2017_774_MOESM15_ESM.pdf]
